# Comparative genomic analysis reveals high intra-serovar plasticity within *Salmonella* Napoli isolated in 2005–2017

**DOI:** 10.1186/s12864-020-6588-y

**Published:** 2020-03-04

**Authors:** Eleonora Mastrorilli, Sara Petrin, Massimiliano Orsini, Alessandra Longo, Debora Cozza, Ida Luzzi, Antonia Ricci, Lisa Barco, Carmen Losasso

**Affiliations:** 1 Istituto Zooprofilattico Sperimentale delle Venezie, Microbial Ecology Unit, Legnaro, Italy; 2 Present address: European Molecular Biology Laboratory, Structural and Computational Biology Unit, Heidelberg, Germany; 3 Istituto Zooprofilattico Sperimentale del Mezzogiorno, Portici, Italy; 4 Istituto Superiore di Sanità, Rome, Italy; 5 Istituto Zooprofilattico Sperimentale delle Venezie, Food Safety Department, Legnaro, Italy

**Keywords:** *Salmonella* Napoli, Comparative genomic analysis, Phylogeny, Accessory genome

## Abstract

**Background:**

*Salmonella enterica subsp. enterica* serovar Napoli (*S*. Napoli) is among the top serovars causing human infections in Italy, although it is relatively uncommon in other European countries; it is mainly isolated from humans and the environment, but neither the reservoir nor its route of infection are clearly defined. This serovar is characterized by high genomic diversity, and molecular evidences revealed important similarities with typhoidal serovars.

**Results:**

179 *S*. Napoli genomes as well as 239 genomes of typhoidal and non-typhoidal serovars were analyzed in a comparative genomic study. Phylogenetic analysis and draft genome characterization in terms of Multi Locus Sequence Typing (MLST), plasmid replicons, *Salmonella* Pathogenicity Islands (SPIs), antimicrobial resistance genes (ARGs), phages, biocide and metal-tolerance genes confirm the high genetic variability of *S*. Napoli, also revealing a within-serovar phylogenetic structure more complex than previously known. Our work also confirms genomic similarity of *S*. Napoli to typhoidal serovars (*S*. Typhi and *S*. Paratyphi A), with *S*. Napoli samples clustering primarily according to ST, each being characterized by specific genomic traits. Moreover, two major subclades of *S*. Napoli can be clearly identified, with ST-474 being biphyletic. All STs span among isolation sources and years of isolation, highlighting the challenge this serovar poses to define its epidemiology and evolution. Altogether, *S*. Napoli strains carry less SPIs and less ARGs than other non-typhoidal serovars and seldom acquire plasmids. However, we here report the second case of an extended-spectrum β–lactamases (ESBLs) producing *S*. Napoli strain and the first cases of multidrug resistant (MDR) *S*. Napoli strains, all isolated from humans.

**Conclusions:**

Our results provide evidence of genomic plasticity of *S.* Napoli, highlighting genomic similarity with typhoidal serovars and genomic features typical of non-typhoidal serovars, supporting the possibility of survival in different niches, both enteric and non-enteric. Presence of horizontally acquired ARGs and MDR profiles rises concerns regarding possible selective pressure exerted by human environment on this pathogen.

## Background

According to data on zoonosis in the European countries [1], *Salmonella* spp. is among the top pathogens causing infections in humans. Although more than 2600 different *Salmonella* serovars have been described to date [2], few of them are responsible for the great majority of human infections [1]. *Salmonella* serovars can be referred to as non-typhoidal or typhoidal, the latter commonly comprising *S*. Typhi and *S.* Paratyphi, being species-specific for humans and causing typhoidal fever. Conversely, non-typhoidal serovars (NTS) are present in different animal reservoirs and are responsible for self-limiting gastrointestinal syndromes.

*Salmonella enterica subsp. enterica* serovar Napoli (*S*. Napoli) is considered a NTS, although it presents low infective dose and prolonged incubation period, together with genetic elements that suggest a close relatedness with typhoidal serovars [3]. Although *S*. Napoli is relatively uncommon in Europe, it is among the top five serovars causing human infections in Italy, with a substantial increase in the number of isolations since 2000 [4, 5]. Moreover, several outbreaks related to this serovar outside of Italy have been linked to the consumption of exported Italian food products (e.g. chocolate bars [6–8], rocket salad [9]). The Rapid Alert System for Food and Feed (RASFF) reported twelve notifications regarding *S*. Napoli to date, all but one involving fresh vegetable products exported from Italy to other European countries (as of July 2019).

*S*. Napoli is generally isolated from humans [10–12], animals (both wild [13–16] and domestic [17]) and the environment [4, 9, 18]. In addition, data from the EnterVet network [19] show that *S.* Napoli is rarely found in farm animals and foodstuff of animal origin [5]. The high frequency of isolation of such serovar from fresh vegetables and during summer season led several authors to speculate that surface water might be a plausible route of contamination [3, 5, 9, 18, 20]. However, up to date, there is no evidence about the definite *S*. Napoli reservoir and its infection route. Moreover, this serovar is characterized by high genomic diversity [9], thus hindering the identification of specific features that could clarify both adaptation to specific environmental/animal reservoirs and its virulence potential.

These evidences led us to perform a comparative genomic study to investigate the genomic potential of *S.* Napoli serovar supporting its ecological and epidemiological success.

## Results

### Data description

The total dataset included 179 *S*. Napoli genomes, of which: 142 newly sequenced, 36 retrieved from Enterobase (www.enterobase.warwick.ac.uk) [21], and 1 from GenBank database (https://www.ncbi.nlm.nih.gov/genbank/) [22]. All strains have been collected in Italy, Germany, Denmark, United Kingdom, Ireland, Poland and the United States, spanning years 2005–2017.

For comparative analysis purposes, we added: 239 Clade A sequences spanning *Salmonella* serovars Typhi, Paratyphi A, Choleraesuis, Newport, Enteritidis, Dublin, Heidelberg, Typhimurium and 1,4, [5],12:i:- and derived from Huedo et al. [3]; 77 Clade B sequences spanning serovars Schwarzengrund, Montevideo, Javiana, Panama, Brandenburg, Miami, Poona, Gallinarum, Pomona, Eastborne, Nottingham, Bredney, Decatur and derived from Didelot et al. [23] and Worley et al. [24]; 1 Clade C sequence belonging to serovar Weslaco and derived from Worley et al. [24]. All the included reference genomes were retrieved from NCBI RefSeq (https://www.ncbi.nlm.nih.gov/refseq/) [25] and GenBank databases (https://www.ncbi.nlm.nih.gov/genbank/) [22].

Additional file 1 reports the complete metadata: serovar, source of isolation, year of isolation, country of isolation, sequence type (ST) for each of the *S.* Napoli collected genomes (sheet 1) and Clade A sequences (sheet 2). Additional file 2 reports the number of *S.* Napoli isolates per source of isolation, isolation year, and country of isolation and ST, respectively.

### Genome sequence similarity and genome annotation

The 178 *S.* Napoli assembly sizes were variable, within a range of [4.41–4.93] Mb, with a GC% content varying between 52.0 and 52.3% (Additional file 1, sheet 1).

The genomic sequence similarity within *S.* Napoli sequences, expressed as the OrthoANI genomic index, resulted in values ranging between 99.50 to 99.99%. MLST analysis divided *S.* Napoli isolates in seven STs. Supplementary Fig. S1 (Additional file 3) reports the minimum spanning tree built using the MLST profile of all *S.* Napoli samples, with circles indicating -as proportions- the different sources of isolation. Genome annotation of *S*. Napoli and Clade A genomes, irrespective of the serovar, resulted in 4066 to 4741 predicted protein-coding sequences, with the overall pangenome including 21,153 genes. A number of 2325 genes were assigned to the Clade A core genome, while 3500 genes were assigned to the core genome of *S.* Napoli only.

### Pangenome analysis and phylogeny

In order to confirm that *S.* Napoli is part of Clade A [3], a SNPs based phylogeny was built including genomes belonging to Clade A, B and C (Supplementary Fig. 2, Additional file 4). To further investigate *S*. Napoli possible relationship with other recombinogenic serovars, we performed a population analysis using STRUCTURE [26], with a cgMLST scheme including 3065 alleles as input. Samples generally clustered in accordance to the above-mentioned SNPs based phylogenetic analysis. Supplementary Fig. 3 (Additional file 5) shows the Maximum Likelihood (ML) best tree built for Clades A, B and C side by side with the population structure identified by STRUCTURE. In every analyzed population (K = 2 to 10), *S*. Napoli samples always grouped together in a single population, with only few samples showing admixture with other population sequences, thus highlighting that *S*. Napoli is not recombinogenic. Once confirmed that *S*. Napoli is part of Clade A, we performed all downstream analysis focusing on Clade A serovars only.

**Fig. 1:**
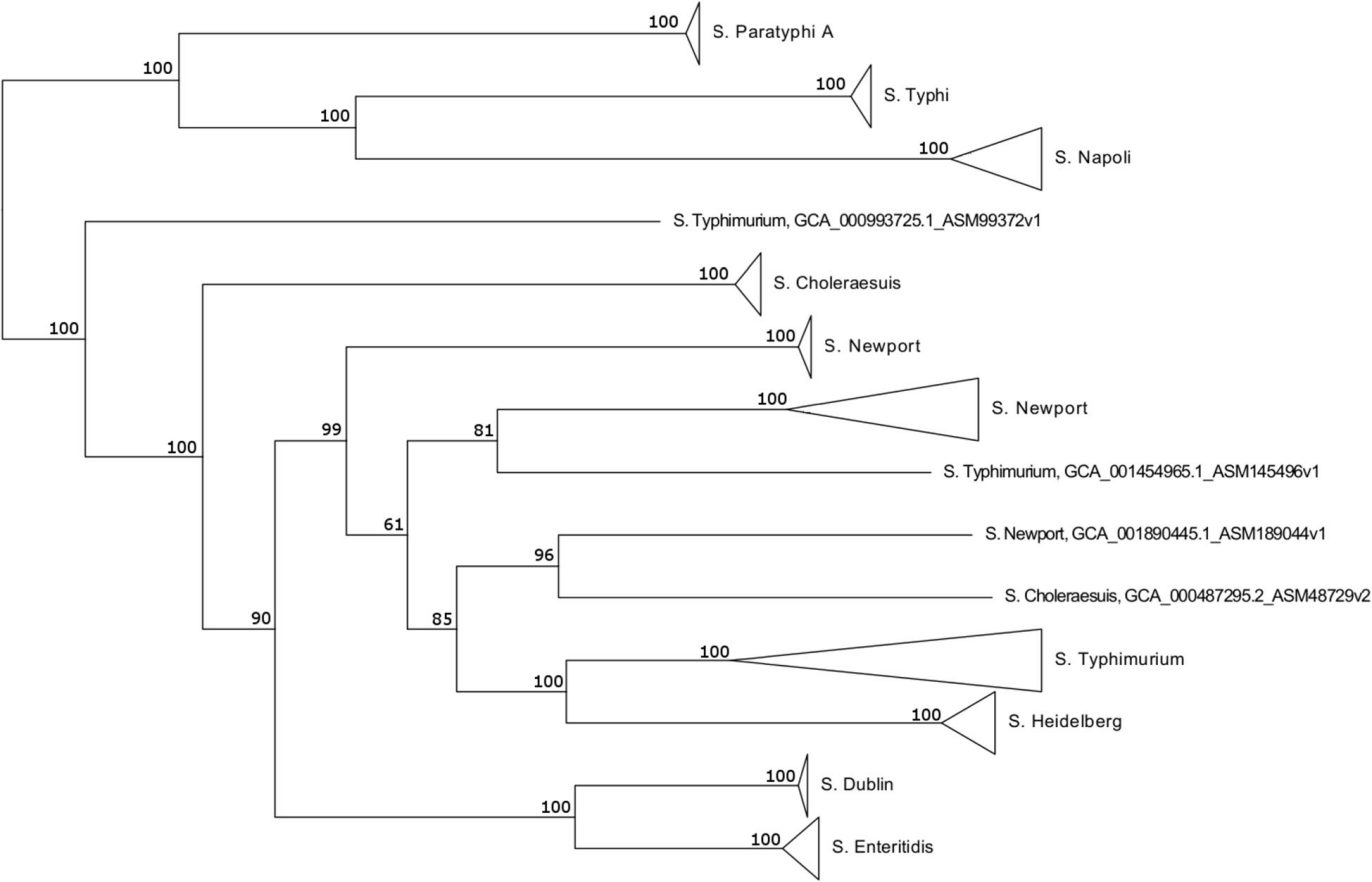
Core genome alignement-based ML phylogeny of all Clade A genomes. Core genome alignment was used for phylogenetic reconstruction using RAxML (version 7.2.8, [27]) with bootstrapping and Maximum Likelihood (ML) search under the GAMMA model of rate heterogeneity. Tree visualization was obtained using FigTree v1.4.4 [28]. Subtrees were collapsed for ease of interpretation, while bootstrap values are indicated as node labels. Subtrees clearly group isolates belonging to the same serovar. *S*. Napoli clusters with typhoidal serovars *S.* Typhi and *S.* Paratyphi A, separately from all other non-typhoidal serovars

**Fig. 2:**
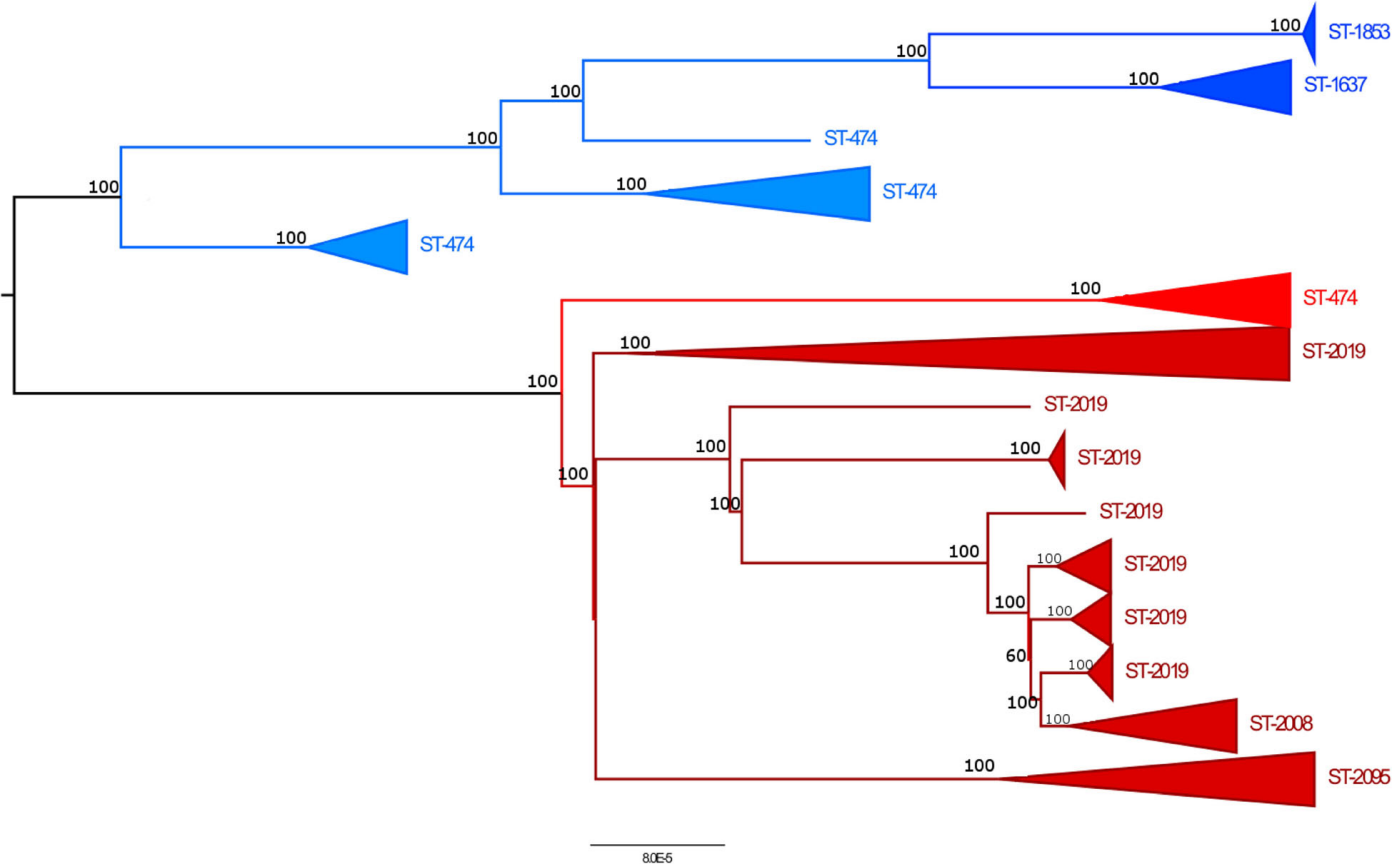
Core genome alignement-based ML phylogeny of *S.* Napoli genomes. Core genome alignment was used for phylogenetic reconstruction using RAxML (version 7.2.8, [27]) with bootstrapping and Maximum Likelihood (ML) search under the GAMMA model of rate heterogeneity. Tree visualization was obtained using FigTree v1.4.4 [28]. Subtrees were collapsed for ease of interpretation, while bootstrap values are indicated as node labels. Subtrees clearly group isolates belonging to the same ST; interestingly, two major clades can be identified (highlighted in blue and red, respectively), both containing isolates belonging to ST-474. This ST, consequently, appears to be biphyletic. Both subclades include also isolates belonging to different STs, other than ST-474, which are non-overlapping between the two subclades. Indeed, the first subclade groups ST-1637 and ST-1853 with ST-474; the second subclade groups ST-2019, ST-2008 and ST-2095 with ST-474

Figure 1 represents the ML best tree built for Clade A serovars, with subtrees collapsed for ease of interpretation; the complete tree is reported in Supplementary Fig. 4 (Additional file 6). *S.* Napoli subtree clustered with *S.* Typhi and *S.* Paratyphi A genomes, forming a distinct clade from the cluster containing all non-typhoidal serovars. Figure 2 represents the ML best tree built for *S.* Napoli samples only; the complete tree is reported in Supplementary Fig. 5 (Additional file 7). Subtrees clearly grouped genomes belonging to the same ST; interestingly, two major clades were identified (Fig. 2), both containing genomes belonging to ST-474. This ST, consequently, appeared to be biphyletic. Both subclades included also isolates belonging to different STs, other than ST-474, which were non-overlapping between subclades. Indeed, the two subclades grouped ST-1637 and ST-1853 with ST-474, and ST-2019, ST-2008 and ST-2095 with ST-474, respectively. A well-defined sub-cluster was highlighted, grouping isolates belonging to ST-1853 and related to a single outbreak occurred in Italy in 2012 and associated to kennel dogs [17].

A Bayesian phylogenetic analysis was performed to estimate *S*. Napoli STs divergence time; a combination of strict molecular clock model and coalescent log normal population size prior was used, since it was significantly better than the other tested models (BF = 276.5). A mean genome-wide corrected evolutionary rate of 8.90 × 10^− 8^ substitution/site/year (credibility interval: 4.87 × 10^− 8^, 13.10 × 10^− 8^) was estimated, indicating that divergence of the two ST-474 clones could be set around 1990 (Supplementary Fig. 6, Additional file 8). Then, around 2000, one of these clones diverged into ST-2019/ST-2095 clone (lower clone in Fig. 2, Supplementary Fig. 5 and Supplementary Fig. 6). From ST-2019, more recently, the ST-2008 and ST-2101 emerged. From the second clone of ST-474, ST-1637 and ST-1853 diverged in the period 2003–2008 (upper clone in Fig. 2, Supplementary Fig. 5 and Supplementary Fig. 6).

### Metadata analyses

ST-474 was found to be temporally the first ST identified in our collection; then ST-1637, ST-2095, ST-2019, ST-2008 emerged. Moreover, ST-474 was always found in each isolation year (Table 1).

**Table 1:** Number of *S*. Napoli genomes per ST and per year of isolation

	YEAR													
		2005	2007	2008	2009	2010	2011	2012	2013	2014	2015	2016	2017	NA
**ST-TYPE**	**474**	✓ (1)	✓ (1)	✓ (1)	✓ (15)	✓ (7)	✓ (5)	✓ (5)	✓ (1)	✓ (7)	✓ (6)	✓ (11)	✓ (4)	✓ (2)
	**1637**		✓ (1)	✓ (2)	✓ (1)	✓ (2)	✓ (5)	–	✓ (1)	–	✓ (1)	✓ (1)	–	✓ (1)
	**2095**			✓ (1)	✓ (1)	–	–	✓ (2)	–	✓ (3)	✓ (8)	✓ (23)	–	✓ (4)
	**2019**					✓ (1)	✓ (2)	✓ (1)	–	–	–	✓ (15)	✓ (1)	–
	**2008**						✓ (1)	–	✓ (1)	✓ (5)	–	✓ (11)	✓ (3)	✓ (4)
	**1853**							✓ (8)	–	–	–	–	–	–
	**2101**											✓ (1)	–	–

*Presence of genome(s) belonging to a specific ST per each year of isolation is indicated by the check mark symbol (✓); number of isolates is reported in brackets. The dash symbol (−) represents missing genomes belonging to each ST after the first year of isolation*

Metadata correlation analysis revealed a statistically significant association between ST and source of isolation (Fisher’s Exact Test for Count Data with simulated *p*-value based on 2000 replicates, two-sided: *p*-value = 0.0004998). The ST vs source association test on partitions of the contingency table (Table 2) highlighted that ST-2095 was significantly associated to human source, while ST-2019 and ST-1637 were significantly associated to the environmental source (Fisher’s Exact Test for Count Data with simulated *p*-value based on 2000 replicates, two-sided: *p*-value: 9.13e-05, *p*-value: 2.92e-05 respectively, after Bonferroni correction for multiple comparison). *S.* Napoli ST-474, however, was the most commonly found ST in our collection, irrespectively of the source of isolation.

**Table 2:** Number of *S*. Napoli genomes per ST and per source of isolation

ST	ANIMALS	FOOD	ENVIRONMENT	HUMAN	Row total
**474**	13	3	12	34	62
**1637**	1	2	8	2	13
**2095**	4	3	2	27	36
**2019**	4	3	7	2	16
**2008**	0	5	8	10	23
Column total	22	16	37	75	150

*Number of available genomes of each ST isolated from each source. All clonal isolates, isolates from unknown or laboratory source were removed; ST-2101 had to be excluded because it was represented by one genome only in our collection*

In addition, combined analysis of phylogeny and metadata revealed that *S.* Napoli phylogenetic clusters were not related to the country nor to the source of isolation. Indeed, samples isolated from environmental, human and animal sources clustered together, thus suggesting that there is no direct relationship between phylogenetic clusters and source of isolation (Additional file 7, Supplementary Fig. S5).

### *Salmonella* Pathogenicity Islands detection

Clade A genomes showed a diverse SPIs profile (see Supplementary Table 3, Additional file 9), including C63PI, SPI-1, SPI-2, SPI-3, SPI-4, SPI-5, SPI-6, SPI-7, SPI-8, SPI-9, SPI-10, SPI-11, SPI-12, SPI-13, SPI-14 and SPI-18. SPIs detection frequencies for each serovar are reported in Table 3. C63PI, SPI-1, SPI-2, SPI-9 were always found in all analyzed genomes.

**Table 3:** Number of genomes containing a hit in the SPI database for each serovar

SEROVAR	*S*. 4, [5],12:i:-	*S*. Choleraesuis	*S*. Dublin	*S*. Enteritidis	*S*. Heidelberg	*S*. Newport	*S*. Napoli	*S*. Paratyphi A	*S*. Typhi	*S*. Typhimurium
**SPI-1/C63PI**	**1**	**3**	**2**	**81**	**27**	**22**	**179**	**7**	**40**	**56**
**SPI-2**	**1**	**3**	**2**	**81**	**27**	**22**	**179**	**7**	**40**	**56**
**SPI-3**	**1**	**3**	**2**	**81**	**27**	**22**	0	6	39	51
**SPI-4**	**1**	**3**	**2**	80	25	21	61	**7**	33	52
**SPI-5**	**1**	**3**	**2**	**81**	**27**	21	**179**	**7**	**40**	**56**
**SPI-6**	0	0	0	0	0	0	0	0	37	0
**SPI-7**	0	0	0	0	0	0	0	0	29	0
**SPI-8**	0	0	0	0	0	0	0	**7**	**40**	0
**SPI-9**	**1**	**3**	**2**	**81**	**27**	**22**	**179**	**7**	**40**	**56**
**SPI-10**	0	0	0	0	0	0	0	0	24	0
**SPI-11**	0	2	0	2	0	1	0	0	0	0
**SPI-12**	**1**	**3**	**2**	79	26	**22**	4	0	**40**	54
**SPI-13**	**1**	**3**	**2**	**81**	**27**	**22**	177	0	0	**56**
**SPI-14**	**1**	**3**	**2**	**81**	**27**	**22**	0	0	0	**56**
**SPI-18**	0	0	0	0	0	0	**179**	**7**	**40**	0

*For each of the investigated SPI sequences, the number of genomes showing a BLAST hit with > 80% identity, > 60% of query coverage and e-value < 0.01 for each serovar is reported. Bold numbers represent cases in which all-available genomes for the serovar have a hit for the corresponding SPI*

*S.* Napoli genomes showed a median number of 6 SPIs per sample, ranging (5-8), which resulted significantly lower compared to all other serovars, that harbored a median of 10 SPIs per sample, ranging (5-12) (Wilcoxon rank sum test with Bonferroni correction, *p*-value < 2.2e-16). Only *S.* Paratyphi genomes showed a comparable number of SPIs to *S.* Napoli (median 7 per sample, ranging (6-7)).

Almost all *S.* Napoli SPIs profiles were characterized by the presence of SPI-5, SPI-13 and SPI-18 and by the absence of SPI-3, SPI-6, SPI-7, SPI-8, SPI-10, SPI-11 and SPI-14. *S.* Typhi and *S.* Paratyphi A genomes, which were characterized by the presence of SPI-8 in place of SPI-13, shared with *S.* Napoli the absence of SPI-14 and the presence of SPI-5 and SPI-18. A complete SPI-18 was found in all *S*. Napoli genomes but two: 16-174481_S8 and 16-174535_S1. In detail, 16-174481_S8 was missing both *hlyE* and *taiA* genes, the two genes included in SPI-18, while 16-174535_S1 was missing *hlyE* gene only. *S.* Napoli shared with all non-typhoidal *Salmonella* SPIs profiles the presence of SPI-13.

Within *S*. Napoli serovar, no unique SPIs profile was identified, although some STs showed a prevalent SPIs profile (e.g. ST-2008, ST-1637 and ST-2095). No link between SPIs profile and phylogenetic clustering, year of isolation or source was identified within *S.* Napoli genomes.

### Acquired antimicrobial resistance genes identification

A significant and strong association was found between serovar and number of genomes harboring acquired ARGs (Pearson’s Chi-squared test, *p*-value < 2.2e-16, Cramer’s V = 0.649) within Clade A. The serovar vs ARGs presence contingency table was partitioned to compare *S.* Napoli vs all other serovars, highlighting that ARGs were detected in fewer *S.* Napoli isolates than in serovar *S.* Typhi, *S.* Heidelberg, *S.* Newport and *S.* Typhimurium (*p*-value = 3.553e-07, *p*-value = 2.2e-16, *p*-value = 2.83e-13 and *p*-value = 8.524e-16, respectively).

However, 5 *S.* Napoli genomes showed at least one acquired resistance gene (ARGs) (Supplementary Table 3, Additional file 9), 4 of which being isolated from human. Moreover, 2 out of 5 genomes displayed a multiresistance profile. In detail, the two multiresistant genomes were ST-474 isolates and harbored a gene of the *aadA* family, conferring resistance to streptomycin (aminoglycosides), together with genes conferring resistance to β-lactams (*bla*), tetracyclines (*tet*) and sulphonamides (*sul*); the 3 genomes showing only one acquired ARG harbored a gene of the *bla* family, conferring resistance to β-lactams.

### Plasmid replicons detection

Twenty-six *S.* Napoli genomes, isolated from environment (*N* = 11), humans (*N* = 11), food (*N* = 1), livestock (*N* = 1), and unknown origin (*N* = 2) showed at least one plasmid replicon (Supplementary Table 3, Additional file 9). Plasmid replicons found in *S.* Napoli isolates belonged to incompatibility groups H (IncHI2A), I (IncI1), F (IncFII, IncFIB), X (IncX), and to plasmid types ColRNAI and Col440l, with plasmid replicon IncFII being the most commonly found. All the *S.* Napoli genomes carrying ARGs showed also IncI, IncF or IncH plasmid replicons in the same genomic region, thus suggesting that ARGs could have been acquired by horizontal gene transfer. *S*. Napoli ST-1853 and ST-2101 did not show any hit in the plasmid replicon database.

Serovar and number of isolates with plasmid replicons showed a strong significant association (Pearson’s Chi-squared test, *p*-value < 2.2e-16, Cramer’s V = 0.56), highlighting that plasmid replicons were detected in fewer *S.* Napoli isolates then in serovars *S.* Enteritidis, *S.* Heidelberg, *S.* Newport and *S.* Typhimurium (*p*-value = 0.0018, *p*-value< 2.2e-16, *p*-value = 0.0011 and *p*-value< 2.2e-16, respectively).

### Antibacterial biocide- and metal-tolerance genes detection

Clade A genomes showed a diverse profile of antibacterial biocide- and metal-tolerance genes (Supplementary Table 3, Additional file 9 and Supplementary Table 4, Additional file 10). The entire dataset was characterized by 123 unique genes; of these 69 were shared by ≥90% of genomes (“core BacMet” hereafter). Additional file 10 summarizes the identified genes in terms of gene name, corresponding BacMet ID, gene product, gene family, targeted biocide or metal compound, gene description, and presence in the core BacMet. When used for hierarchical clustering (Supplementary Fig. 7, Additional file 11), BacMet profiles divided sequences into two main subgroups: one including *S.* Napoli, Typhi and Paratyphi A genomes; the other one grouping all the non-typhoidal genomes. *S.* Napoli shared with *S.* Typhi and *S.* Paratyphi A the absence of several operons: *ges, gol*, *mer, oqx*, *pco*, *sil* and *ter*, conferring tolerance to gold, mercury, quaternary ammonium compounds, copper, silver and tellurium, respectively.

Moreover, *S.* Napoli genomes showed a median number of hits in the BacMet database significantly lower if compared to non-typhoidal genomes. In detail, *S.* Napoli genomes carried less antibacterial biocide- and metal-tolerance genes than *S.* Choleraesuis, Enteritidis, Heidelberg, Newport and Typhimurium genomes (Wilcoxon rank sum test with Bonferroni correction, *p*-value = 0.001348, *p*-value < 2.2e-16, *p*-value < 2.2e-16, *p*-value = 9.217e-13 and *p*-value < 2.2e-16, respectively).

### Pangenome functional annotation

The pangenome was functionally annotated and a COG (Clusters of Orthologous Groups) category was assigned to 8847 genes out of 21153 constituting the pan genome (41.82%).

**Fig. 3:**
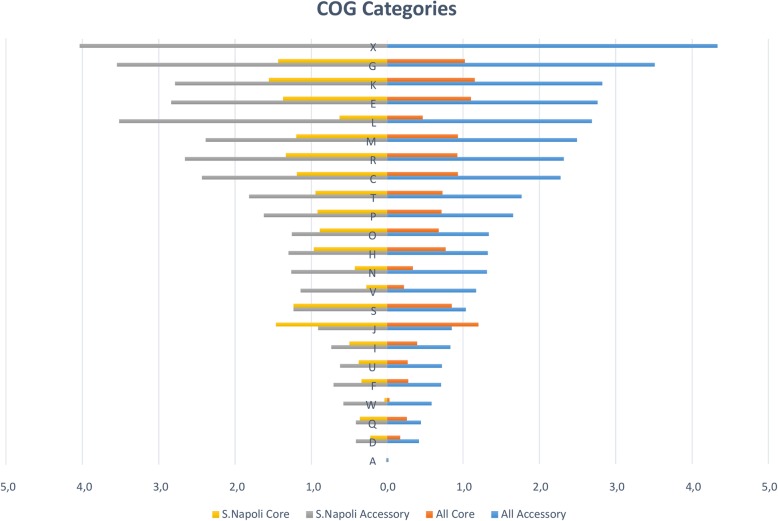
COG category distribution within *S.* Napoli genomes vs all Clade A genomes. Distribution of COG categories in *S.* Napoli (left panel): core genes (yellow) and accessory genes (gray). Distribution of COG categories in the entire *Salmonella* dataset (right panel): core genes (orange) and accessory genes (blue). Results are reported after genes with no COG category assigned were removed. x-axis represents the percentage of genes belonging to each category. Among the categorized genes, the *S.* Napoli core genome dataset shows several categories having a similar occurrence, with none of them dominating over the others. Conversely, the *S.* Napoli accessory genome is largely composed by genes, whose products could be assigned to the X (Mobilome: prophages, transposons), G (Carbohydrate transport and metabolism) and L (Replication, recombination and repair) COG categories. These results suggest that the accessory genome comprises both mobile elements, often involved in virulence/pathogenicity, and genes involved in core metabolic functions and defense mechanisms. No relevant difference in COGs occurrence distribution can be highlighted when comparing the* S.* Napoli dataset and the entire datatset: similar patterns can be found both in core- and accessory- genome COG frequency distribution

*S.* Napoli accessory genome was largely composed by genes, whose products can be assigned to the X (Mobilome: prophages, transposons), G (Carbohydrate transport and metabolism) and L (Replication, recombination and repair) COG categories (Fig. 3). Interestingly, when comparing the accessory/core occurrence ratio, the X (Mobilome: prophages, transposons) and V (defense mechanisms) categories were more frequently annotated in the accessory genome. These results suggest that the accessory genome comprised both mobile elements, often involved in virulence/pathogenicity, and genes involved in core metabolic functions and defense mechanisms. No relevant difference in COGs occurrence distribution was highlighted when comparing *S.* Napoli genomes to the entire Clade A dataset (Fig. 3).

Similarly, both core and accessory genomes were annotated according to the Gene Ontology categories. At least a GO (Gene Ontology) term was assigned to 9935 genes out of 21153 constituting the pan genome (46.96%). In Additional file 12, the top 10 enriched terms for each GO category are reported, for *S.* Napoli vs the whole Clade A dataset. Interestingly, among Biological Processes (BP) *S.* Napoli genomes were characterized by terms suggesting mechanisms of interaction with hosts and virulence-related processes; conversely, the Clade A dataset showed enrichment in more general terms involved in DNA metabolism and processing involving mobile genetic elements.

Accessory genome could also be divided into several groups: 2590 genes were found within *S.* Napoli genomes only, 526 genes were found within typhoidal isolates only (*S.* Typhi and *S.* Paratyphi A), and 3181 genes were found within non-typhoidal Clade A isolates (Additional file 13). Accessory genes found within *S.* Napoli genomes and typhoidal genomes were characterized by a high number of phagic/prophagic proteins as well as transposons and integrases, while accessory genes found within non-typhoidal isolates only were characterized by a diverse set of metabolic functions. Genes shared among all *S.* Napoli STs belonged to basic and vital COG categories, such as G (Carbohydrate transport and metabolism), E (Amino acid transport and metabolism) and K (Transcription).

Among all ST-unique genes, i.e. genes not shared between different STs, several belonged to G (Carbohydrate transport and metabolism) and E (Amino acid transport and metabolism) COG categories. ST-1637 and ST-2019 were characterized by genes belonging to COG category N (Cell motility), mainly fimbrial proteins, outer membrane usher proteins and chemotaxis proteins. ST-2095 and ST-474 were characterized by genes belonging to COG category X (Mobilome: prophages, transposons). ST-1637 only was characterized by genes belonging to COG category T (Signal transduction mechanisms), including sensor proteins and chemotaxis proteins. ST-1853 only was characterized by genes belonging to COG category V (Defense mechanisms), including restriction-modification system proteins.

### Pseudogene identification

Both the total amount of detected pseudogenes and their percentage with respect to the total annotated CDSs are reported in Supplementary Fig. 8 (Additional file 14). Kruskal-Wallis rank sum test confirmed a significant difference in total pseudogene number distribution among different serovars (*p*-value < 2.2e-16). The number of pseudogenes detected in *S*. Napoli was significantly lower if compared to serovars known to be host-restricted as *S*. Gallinarum (adjusted *p*-value = 5.143517e-04), *S*. Typhi (adjusted *p*-value = 2.281705e-19), and *S*. Paratyphi A (adjusted *p*-value = 4.411131e-04). Indeed, the number of pseudogenes detected in *S*. Napoli genomes was comparable to that reported for other serovars known to have a broad host range, such as *S*. Enteritidis and *S*. Typhimurium.

### Intact prophage detection

A total of 42 lysogenic prophage sequences were detected in our dataset (Additional file 15). Globally, *S.* Napoli isolates reported a significantly lower number of prophages than other serotypes (Wilcoxon rank sum test, *p*-value < 2.2e-16). In Supplementary Fig. 9 (Additional file 16) prophage sequences detected in more than one sample (*n* = 35) were reported in a heatmap.

P88 prophage was found in 45 *S.* Napoli genomes, thus resulting the most frequently detected prophage in this serovar. Although it was not detected in all *S.* Napoli genomes, it was found in genomes belonging to all STs excluding ST-2095. Moreover, this prophage was found only in a few other genomes belonging to *S.* Typhi, *S.* Paratyphi A and *S*. Enteritidis serovars. Gifsy-1 and Gifsy-2 were absent in *S*. Napoli genomes but, as expected, they were found in almost all *S.* Newport and Typhimurium genomes, and in all *S.* Heidelberg, Dublin and Enteritidis genomes. No multiple copies of intact prophage sequence were detected in the entire dataset.

## Discussion

Here, we compared 179 *S*. Napoli genomes with 239 typhoidal and non-typhoidal sequences belonging to serovars *S*. Typhi, Paratyphi A, Choleraesuis, Newport, Enteritidis, Dublin, Heidelberg, Typhimurium and 1,4, [5],12:I, thus enabling us to present the largest comparative genomic study for *S.* Napoli. Huedo et al. [3], analyzing a limited number of genomes over a subset of core genes, placed *S*. Napoli in the same subclade as *S*. Typhi and *S*. Paratyphi A serovars. Here, the high number of *S*. Napoli genomes collected allowed us to get insight into the phylogeny and genomic asset of this highly diverse [29] serovar. As expected, phylogenetic analysis was consistent with previously published data [24, 30, 31], confirming that: (i) *S*. Napoli is part of the Clade A; (ii) it clusters with typhoidal *Salmonella* serovars [31]; (iii) it appears to be a monophyletic sub-clade. In accordance to that, *S*. Napoli isolates do not show recombination with other serovars.

Both the genomic and the phylogenetic analyses highlighted high intra-serovar variability within *S*. Napoli isolates. This high genomic diversity is coherent with the difficult identification of a specific niche for this serovar [4, 5, 9], as it was previously reported in literature for other promiscuous serovars [32, 33].

*S*. Napoli phylogeny highlighted that sequences mainly cluster by ST. Interestingly, both ML and Bayesian analysis support the hypothesis that ST-474 is biphyletic and that it could be the common ST ancestor for all the other STs identified [24, 34].

Metadata integration allowed us to point out a statistically significant association between ST and source, indicating that ST-2095 was more frequently isolated from human samples, while ST-1637 and ST-2019 were more frequently isolated from the environment. Previous studies suggested that the environment, and specifically surface waters, could play an important role in the diffusion of *S*. Napoli [4, 5, 9, 18].

Interestingly, ST-1637 and ST-2019, found to be associated to environmental sources, were the only STs showing a high number of genes coding for cell motility proteins, as well as sensor proteins. These features might be favoring the ability of these STs to sense the surrounding environment and eventually activated motility mechanisms in response to different *stimuli*. Moreover, ST-1853 was characterized by showing an unshared set of defense mechanism proteins, including restriction-modification system proteins, which are known to be employed by bacteria in natural genetic transformation by recombination [35]. Noteworthingly, ST-474 and ST-2095, which were the most frequently isolated STs as well as two of the STs that appeared earlier within our collection, were characterized by displaying the mobilome as major COG category and a high number of unique genes annotated as prophagic genes or transposons, thus suggesting that both carried a set of features favoring genome plasticity.

According to our data, *S.* Napoli serovar is characterized by the frequent identification of P88 phage, which is reported to be phylogenetically divergent from other P2-like prophages [36]. Moreover *S.* Napoli genomes are characterized by the absence of many prophage sequences that are commonly identified in *S. enterica* such as Gifsy-1 and Gifsy-2 [37]. Previous studies have suggested that prophage sequences are a great sources of genomic variability in *S. enterica*, since they provide sources of virulence determinants and can be acquired by horizontal gene transfer [38]. However, no definitive prophage signature could be assigned to *S.* Napoli serovar, although we cannot exclude prophage underestimation due to fragmentation of de novo assembly.

Concerning SPIs, they appeared highly heterogeneous and not related to ST, source or year of isolation. SPI-18, firstly described in *S*. Typhi [39], was identified in every *S*. Napoli genome in our collection. This pathogenicity island includes two ORFs, corresponding to *hlyE* gene encoding for a pore-forming hemolysin, and *taiA* gene, which encodes for a *S*. Typhi-associated invasin [40]. *hlyE* gene has been found also in the human specific typhoidal serovars *S*. Paratyphi A [41] and, despite having a pore-forming activity, the protein does not display hemolytic activity on blood agar plates, but it is able to lyse epithelial cells [42]. Moreover, Fuentes and colleagues [39] demonstrated the contribution of *hlyE* gene to the ability of *S*. Typhi to enter human epithelial cells in vitro. Interestingly, SPI-18 has been detected also in other different serovars, namely *S*. Brandenburg [43], *S*. Montevideo [44], *S*. Panama and *S*. Schwarzengrund [45]. The presence of SPI-18 in *S*. Napoli genomes supports its affinity to typhoidal serovars, as already suggested by Huedo et al. [3].

Conversely, the presence of SPI-13 suggests some persisting affinity of *S.* Napoli to non-typhoidal serovars; this SPI, indeed, is present in different *S. enterica* serovars, including *S*. Enteritidis, Typhimurium, Cholerasuis and Gallinarum, but it is absent in *S*. Typhi and *S*. Paratyphi A, which instead carry SPI-8 in the same genomic location [46–48]. Espinoza et al. [49] showed that SPI-13 is required for internalization of *S.* Enteritidis in murine macrophages, but not human macrophages due to a lower amount of itaconate produced by human macrophages in response to bacterial infections.

Since the presence of different SPIs ensures the accumulation of different virulence mechanisms [50], the evidence of co-existence of SPI-18 and SPI-13 in *S.* Napoli genomes strengthens our speculation that this serovar could have developed a peculiar genomic asset, that allows it to invade a wider range of hosts and environments. This was confirmed by metadata analyses and pseudogene content that was found comparable to *S*. Enteritidis and *S*. Typhimurium, which are known to have a wide host range [37].

Concerning the presence of biocide- and metal-tolerance genes, *S.* Napoli, *S*. Typhi and *S*. Paratyphi A are characterized by a complex deletion pattern, including the *mer*, *oqx*, *pco*, *sil* and *ter* operons. Although many different gene sets may concur to antibacterial biocide- and metal-tolerance [51, 52], such pattern reveals that *S.* Napoli may be less adapted to survive in biocide- or metal- enriched environments, such as farming environment. Such observation is coherent with our sampling frequencies as well as previous studies that rarely found *S.* Napoli in domestic or farmed animals [18, 29, 53]. Moreover, although a prevalent biocide- and metal-tolerance gene profile exists among the investigated genomes, *S.* Napoli showed a diverse set of profiles, highlighting once again that this serovars displays high intra-serovar genomic variability for non-core functions.

Due to the importance of horizontal gene transfer in the spread of drug resistance and virulence factors [54, 55], we also investigated acquired ARGs and plasmid replicons in our *S*. Napoli genomes. Over 179 sequences, only five isolates showed ARGs, conferring resistance to β-lactams, tetracyclines, sulphonamides and aminoglycosides, thus confirming a general level of pansusceptibility within *S*. Napoli serovar [9]. Interestingly, two out of five isolates showed multidrug resistance (MDR) profiles. Recently, resistance to aminoglycosides due to the presence of *aadA25* gene [10], and resistance to third-generation cephalosporins due to the presence of *bla*_CTX-M-1_ [11, 12] and *bla*_CTX-M-15_ genes (Petrin et al. 2019, submitted) were reported. To the best of our knowledge, this is the first time that *S*. Napoli resistant isolates not belonging to ST-474 (i.e. ST-2008 and ST-2095) are identified. Additionally, for the first time as well, in the present work MDR profiles in serovar Napoli are described. In addition to that, four out of five resistant isolates in our collection are of human origin, and with ARGs location of conceivably plasmidic origin, given the contextual presence of plasmid replicons. Altogether, these evidences led us to speculate that only few *S.* Napoli clones, circulating in human hosts, might be subjected to selective pressure leading to horizontal acquisition of ARGs.

As for plasmid replicons, only 26 samples showed at least one replicon, therefore we speculate that *S*. Napoli serovar hardly acquires genes by means of horizontal gene transfer (unless the existence of a selective pressure favors it) or that maintaining plasmids is not convenient for the fitness of *S*. Napoli. Indeed, *bla*_CTX-M-15_ determinants have extensively been associated with F-type plasmids [56], and with IncI1 plasmids [57], where they are nowadays recognized among the major vehicle for dissemination of extended-spectrum β-lactamases (ESBLs). Multidrug resistance is associated also with IncHI2 plasmids, also found in our samples, in which sulphonamides, tetracyclines, aminoglycosides and streptomycin resistance genes are also commonly encoded [58]. Interestingly, IncHI2 plasmids show optimal transfer by conjugation at temperatures between 22° and 30 °C, thus suggesting possible spread of resistance in the environment [59–61].

Possibly, the high diversity found in *S.* Napoli serovar supports its high adaptability to different selective pressure conditions: from wild environments, where sensing and mobility are enforced but isolates are still pansusceptible, to human environment, where the acquisition of plasmids and antimicrobial resistances may be advantageous. Although not conclusive on investigating *S.* Napoli genomic diversity, these evidences should testify the need of attention for this serovar from healthcare providers, also outside Italian borders.

We cannot exclude, however, that our results might be hindered by our data collection protocol. Our dataset suffers indeed from a collection bias due to voluntary submission of data. This bias, which may have impacted the proper estimation of the frequency of isolation of *S*. Napoli from different sources and countries, has to be ascribed to the absence of an EU target control plan for *S*. Napoli, evenly spanning both years/sources of isolation.

## Conclusion

The availability of large whole genome sequences datasets greatly contributes to improve the knowledge on the genomic content of pathogens of interest for public health. In the specific case of *S*. Napoli, our study aimed at shedding light on the genomic asset of this successful serovar. This study performed comparative genomic analysis of an unprecedently met number of *S.* Napoli genomes, and investigated its possible relationship with both typhoidal and non-typhoidal *Salmonella* serovars by phylogenetic and pangenome analyses. Results definitively clarified that this iNTS shares genome similarity with typhoidal serovars, although preserving several mechanisms common to non-typhoidal serovars. These features, together with the evidence of its high genomic plasticity, support its ability to colonize different niches, from humans, to animals and the environment.

## Methods

### Sample collection and whole genome sequencing

As part of the ENGAGE consortium (http://www.engage-europe.eu/), a total number of 140 Italian S. Napoli isolates were collected and sequenced. The Italian Reference Laboratory for Salmonellosis at Istituto Zooprofilattico Sperimentale delle Venezie (IZSVe) provided 32 isolates and acted as a hub for the Italian *S*. Napoli isolates, with 40 strains contributed from Istituto Superiore di Sanità (ISS) and 68 contributed from Istituto Zooprofilattico Sperimentale del Mezzogiorno. Moreover, 16 *S*. Napoli sequences from other European countries, all spanning years 2005–2017, were obtained: the German Federal Institute for Risk Assessment (BfR) provided 11 sequenced genomes, and Technical University of Denmark (DTU) provided 5 sequenced genomes (Additional file 1, Sheet1).

Italian strains were recovered from stock culture conserved at − 80 °C and serotyped according to White-Kaufmann-Le Minor scheme [2] with the traditional slide agglutination method for serotype confirmation.

Pure colonies of *S.* Napoli isolates were cultured on TA (tryptose agar) plates and incubated at 37 °C for 24 h. Genomic DNA (gDNA) was extracted using a commercial column-based protocol (QIAamp DNA Mini, QIAGEN, Valencia, CA), and purified gDNA was quantified with a Qubit 3.0 Fluorometer (Life Technologies). Libraries for whole genome sequencing were prepared using a Nextera XT DNA sample preparation kit (Illumina). High-throughput sequencing was performed with MiSeq Reagent kit v3, resulting in 251-bp-long paired-ends reads. Sample quality was assessed via FastQC v0.11.2 [62] and sample reads were trimmed for both quality and length using Trimmomatic 0.32 [63] with the following options: removal of Nextera adapters sequences; cut bases off the start of the read, if below a quality score of 20; cut bases off the end of the read, if below a quality score of 20; sliding window trimming, clipping the read once the average quality within the window (4 bp) falls below 20; finally, drop the read if it is shorter than 100 bp. Subsequently, reads were de novo assembled using Spades 3.10.1 [64] and the quality of assembly was assessed using QUAST 3.1 [65].

After *in-silico* serotyping, 14 *S*. Napoli genomes were excluded because serotype was not confirmed (data not shown). Table 1 reports the final number of genomes available for subsequent analyses.

### Sequences retrieval

GenBank [22] and RefSeq [25] were accessed to retrieve: 239 Clade A sequences spanning *Salmonella* serovars Typhi, Paratyphi A, Choleraesuis, Newport, Enteritidis, Dublin, Heidelberg, Typhimurium and 1,4, [5],12:i:- and derived from Huedo et al. [3]; 77 Clade B sequences spanning serovars Schwarzengrund, Montevideo, Javiana, Panama, Brandenburg, Miami, Poona, Gallinarum, Pomona, Eastborne, Nottingham, Bredney, Decatur and derived from Didelot et al. [23] and Worley et al. [24]; 1 Clade C sequence belonging to serovar Weslaco and derived from Worley et al. [24]. For each reference genome both chromosome and plasmids scaffolds (when available) were retrieved separately.

All *S.* Napoli sequence data available on Enterobase [21] (accessed on 26/02/2018) were recovered (*N*= 47). Eleven of them were excluded because they were corresponding to internally sequenced genomes.

### Strain typing

*In-silico* serotyping was performed using three different tools: MOST 1.0 [66] and SeqSero 1.0 [67] on raw data, and SISTR 1.0.2 [68] on assembled data. Results were compared, and only samples being *in-silico* serotyped as *S.* Napoli by at least two out of three tools were subjected to downstream analyses. Then, each genome was subjected to *in-silico* sequence typing using the MLST scheme for *Salmonella enterica* and the MLST database (downloaded on 05/03/2018) [69] for BLAST search (version 2.7.1, [70]).

### Genome annotation, pangenome analysis and phylogeny

In order to elucidate whether or not *S.* Napoli confirms to be part of Clade A, we performed a core SNPs phylogenetic analysis including a number of 78 genomes from serovars in Clade B (*S*. Schwarzengrund (*n*=3), *S*. Montevideo (*N*=21), *S*. Javiana (*N*=2), *S*. Panama (*N*=1), *S*. Brandenburg (*N*=3), *S*. Miami (*N*=6), *S*. Poona (*N*=1), *S*. Gallinarum (*N*=4), *S*. Pomona (*N*=1), *S*. Eastborne (*N*=6), *S*. Nottingham (*N*=25), *S*. Bredney (*N*=2), *S*. Decatur (*N*=2)) and Clade C (*S*. Weslaco (*N*=1)). A concatenation of SNPs was obtained using kSNP3 3.0 [71], with a k-mer length of 21 as suggested by Worley et al. [24]. The phylogenetic tree was derived using FastTree [72], with default parameters for nucleotide input (GTR-GAMMA model of nucleotide substitution).

For further comparison, all Clade A genomes (both the downloaded reference genomes and the assembled ones) were automatically annotated using Prokka (version 1.11, [73]) and subjected to core and accessory genome extraction using Roary (version 3.6.2, [74]). Core genome alignment was used for phylogenetic reconstruction using RAxML (version 7.2.8, [27]) with bootstrapping and Maximum Likelihood (ML) search under the GAMMA model of rate heterogeneity. Tree visualization and annotation were obtained using FigTree (version 1.4.4 [28]). Both core alignment and phylogenetic reconstruction were separately performed within *S.* Napoli genomes only and within the Clade A genomes collection.

For deeper investigating of *S*. Napoli population, a core-, nonhomoplastic- SNP matrix was generated using kSNP3 3.0 [71] with a k-mer length of 21 [24]. The core-SNPs matrix was used to calculate the evolutionary rate of *S.* Napoli STs testing two molecular clocks (strict and relaxed log normal) and two demographic models (constant population size and log normal expansion) using BEAST v1.8 [75] under 50 million generations (sampling every 5000), with posterior probability as statistical support for each clade. Tracer 1.7 software [76] was used to assess convergence of the MCMC (Markov Chain Monte Carlo) chains on the basis of the effective sampling size (ESS ≥ 200) after a 10% burn-in. Bayes factor (BF) test was used for best fitting model selection [75]. Only values of 2lnBF ≥ 5 were considered significant [75]. The trees were summarised in a maximum clade credibility (MCC) target tree using the Tree Annotator program after a 10% burn-in. Clock rate and time of the most recent common ancestor (MRCA) estimates were scaled to genome-wide units of substitution per site per year considering the genome size of the only complete *S*. Napoli genome (NZ_CP030838, 4,679,033 nucleotides).

### Population recombination analysis

A core-genome MLST (cgMLST) matrix, obtained using chewBBACA [77] with default parameters and trained with *Salmonella enterica* gene prediction model file, was used as input for STRUCTURE 2.3.3 [26]. The cgMLST was built for all the genomes included in Clade A, B and C. The tool run for 50,000 replicates (burn-in: 10000 replicates), assuming a population size ranging from 2 to 10.

### Genome sequences similarity

Overall genome relatedness index, measured as the Orthologous Average Nucleotide Identity (OrthoANI) was determined by using the OrthoANI tool [78].

### Metadata analyses

Metadata (Source of isolation, Collection Year, Country of Isolation, Serovar and Sequence Type) were analyzed for correlation/independence using the *corr.test* function in R (as implemented in the *psych* package [79]). Moreover, all metadata were analyzed for association using Fisher’s exact test or Pearson’s Chi-squared test (according to the expected frequencies of the contingency table cells) as implemented in the *fisher.test* or *chisq.test* function in R (from the *stats* package [80]), respectively. Association test results were supported by Cramer’s V association value as implemented in the *assocstat* function in R (from the *vcd* package [81]). Results were considered statistically significant when *p*-value< 0.05, using Bonferroni correction for multiple testing when needed. Moreover, metadata were used to examine phylogeny results and check for possible associations between sample clustering and any metadata.

### Plasmids replicons, *Salmonella* Pathogenicity Islands and acquired antimicrobial resistance genes search

Contigs were searched for plasmid replicons using blastn (version 2.7.1, [70]) against PlasmidFinder 1.3 database (downloaded on 05/03/2018) [82]; for *Salmonella* Pathogenicity Islands (SPIs) against SPIFinder 1.0 database (https://cge.cbs.dtu.dk/services/SPIFinder/, downloaded on 05/03/2018); for acquired antimicrobial resistance genes (ARGs) against ResFinder 3.0 database (downloaded on 05/03/2018) [83]. Finally, contigs were searched for antibacterial biocide- and metal- tolerance genes using blastx (version 2.7.1, [70]) against BacMet database (downloaded on 12/03/2018) [84]. E-value thresholds were adjusted for each search depending on database size and were set as follows: 0.001 for plasmid replicons search, 0.01 for SPIs search, 0.01 for ARGs search, 1e-4 for BacMet search, respectively. All hits were required to have a 60% minimum coverage of the reference sequence found in the database, while the minimum required percentage of identity was 90% for plasmid replicons search, 80% for SPIs search, 80% for ARGs search, and 90% for the BacMet search. SPI-18, which was not included in the SPIFinder database at the time of download, was manually retrieved from *S.* Typhi str. CT18 reference genome (NCBI accession number NC_003198.1, nucleotide positions 1,455,055–1,456,801) [40], as it was previously described as characterizing *S.* Napoli as well as typhoidal serovars [3]. All results were filtered so that when multiple hits occurred in the same contig and position, only the hit with maximum bitscore was retained.

BacMet hits were used to create a profile according to the presence/absence of the genes included in the database. Such presence/absence profiles where grouped by hierarchical clustering based on Jaccard distance [85] and Ward agglomeration method [86, 87]. Moreover, core BacMet profiles were identified as genes present in at least 90% of the investigated samples.

### Pangenome functional annotation

Gene presence/absence within the entire sample set was exported; firstly singletons (i.e. genes found in one sample only) were removed, then data were divided into core genome (i.e. genes found in 99 to 100% of samples) and accessory genome (i.e. all genes not belonging to the core genome).

Core- and accessory genome genes were characterized in terms of COG (Clusters of Orthologous Groups of Proteins) [88]. The functional annotation of the translated gene sequences was performed with the NCBI Batch CD-Search tool [89] to identify conserved domains by using a mirrored COG database with the default settings (e-value equal to 0.01, a maximum number of hits of 500, and composition-corrected scoring turned on). COGs were then sorted by categories in descending order, to identify the COGs more frequently annotated in the core genome. For all those genes that were annotated with more than one COG, each of the different reported COGs was considered for the frequency computation.

Similarly, both core and accessory genome were annotated for the GO (Gene Ontology) [90] terms in the three categories (BP – Biological Process, CC – Cellular Component and MF – Molecular Function) by blasting them against Uniprot Bacteria [91] and assigning the ontology only if the best match showed both coverage and similarity higher than 80%.

Accessory genome was also explored to extract genes found within *S.* Napoli isolates, genes found within typhoidal isolates (*S.* Typhi and *S.* Paratyphi A) and genes found within non-typhoidal Clade A isolates (*S.* Choleraesuis, Newport, Enteritidis, Dublin, Heidelberg, Typhimurium and 1,4, [5],12:i:-). Each of these groups of genes were characterized in terms of most frequent COGs. Within *S.* Napoli isolates, accessory genome was also explored to extract genes found within each sequence type (ST), and each of these groups of genes were characterized in terms of most frequent COG.

### Pseudogenes identification

Pseudogenes were detected using DFAST v1.2.4 [92] with default parameters. This software has a specific routine for pseudogenes detection, returning frameshifted CDSs or CDSs containing internal stop codons. Dunn test of multiple comparisons was used following a significant Kruskal-Wallis test, with a Benjamini-Hochberg adjustment to control the FDR, to assess the significant difference among pairs of serovars [93].

### Intact prophages search

Prophages sequences were detected in both assemblies and downloaded sequences using PHASTER [94], throughout the API (Application Programming Interface). Json files returned from the server were then parsed by an *in-house* python script to convert them in tabular format; only “intact” prophages were considered for downstream analysis.

## Supplementary information

**Additional file 1: **Tabular data. Metadata of included genomes. Additional file 1 reports the sample name, organism name, source of isolation, isolation year, country of isolation, sequence type (ST) for each of the included genomes. Origin details whether the genome was derived from the ENGAGE consortium, from Enterobase/GenBank/RefSeq database. Summary metrics (total sequences, GC%, total length, number of contigs, N50, number of CDS) are also reported. Supplementary Table 1 (“S_Napoli genomes” sheet) details info for *S.* Napoli collected isolates, Supplementary Table 2 (“Other serovar genomes” sheet) details info for the downloaded reference genomes belonging to Clade A.

**Additional file 2: **Tabular data. Pivot table of sample metadata. Number of *S.* Napoli isolates per source of isolation, isolation year, country of isolation and ST, respectively.

**Additional file 3: Figure. S1.** MLST minimum-spanning tree of all *S.* Napoli genomes. Core genome MLST profiles of all *S.* Napoli isolates was used to build a minimum spanning tree with pie charts describing sources of isolation found for each ST. *S*. Napoli shows a high variability of sources contributing to each ST, with the exception of ST-1853, related to a single outbreak occurred in Italy in 2012 and associated to kennel dogs [17], and ST-2101 that comprises one sample only. Hs: *Homo sapiens*, EN: Environment, FO: Food, CA: Companion Animal, LI: Livestock, PO: poultry, WA: Wild Animal, LA: laboratory.

**Additional file 4: Figure S2.** Core genome alignment-based ML phylogeny of *Salmonella* serovars belonging to Clade A, B, and C. Core genome alignment was used for phylogenetic reconstruction kSNP3 3.0 [71], with a k-mer lenght of 21. The phylogenetic tree was derived using FastTree [72], with default parameters for nucleotide input (GTR-GAMMA model of nucleotide substitution). Subtrees were collapsed for ease of interpretation. *S.* Napoli clusters within Clade A, in the typhoidal subclade.

**Additional file 5: Figure S3.** Population recombination analysis of serovars belonging to Clade A, B, and C. Core-genome MLST (cgMLST) matrix, including 3065 alleles, trained with *Salmonella enterica* gene prediction model file, was used as input for STRUCTURE 2.3.3 [26]. A model-based Bayesian clustering method was used to cluster samples into groups. Samples generally clustered in accordance to the phylogenetic analysis (Fig. S2). Interestingly, in every analyzed population (K = 2 to 10), *S*. Napoli isolates always group together in a single population, thus highlighting that *S*. Napoli isolates do not show recombination with other serovars.

**Additional file 6: Figure S4.** Core genome alignement-based ML phylogeny of all genomes belonging to Clade *A. core* genome alignment was used for phylogenetic reconstruction using RAxML (version 7.2.8, [27]) with bootstrapping and Maximum Likelihood (ML) search under the GAMMA model of rate heterogeneity. Tree visualization was obtained using FigTree v1.4.4 [28]. *S*. Napoli clusters with typhoidal serovars *S.* Typhi and *S.* Paratyphi A, separately from all other non-typhoidal serovars.

**Additional file 7 Figure S5.** Core genome alignement-based ML phylogeny of *S.* Napoli genomes. Core genome alignment was used for phylogenetic reconstruction using RAxML (version 7.2.8, [27]) with bootstrapping and Maximum Likelihood (ML) search under the GAMMA model of rate heterogeneity. Tree visualization was obtained using FigTree v1.4.4 [28]. Within *S.* Napoli serovar, samples cluster primarily by ST, and divide into two major subclades. No significant correlation could be found between phylogenetic clustering and source of isolation. Here source of isolation is shown as a colour scale.

**Additional file 8 Figure S6.** Bayesian phylogenetic analysis. *S*. Napoli STs divergence investigation was performed using BEAST [75]. The combination of strict molecular clock model and coalescent log normal population size prior was used. A mean genome-wide corrected evolutionary rate of 8.90 × 10–8 sub/site/year (credibility interval: 4.87 × 10–8, 13.10 × 10–8) was estimated starting from a 19,496 SNPs matrix built with kNP3 3.0 [71] and indicates that two ST-474 clones might have diverged around 1990. The branches of the maximum clade credibility (MCC) tree are color-coded for comparability with Fig. 2. The scale at the bottom of the tree correspond to calendar years.

**Additional file 9: **Tabular data. Genomic characterization summary table. Supplementary Table 3, Additional file 8 summarizes all information derived from the genomic characterization of the genomes, including: ST, subclade (A or B, for *S.* Napoli genomes only), plasmid replicons, antimicrobial resistance genes (AMR), SPI profiles and BacMet profiles. Additionally, it also reports basic metadata as collection year, country of isolation, source of isolation and serotype to ease data investigation.

**Additional file 10 **Tabular data. BacMet hits description. Additional file 9 summarizes the BacMet genes identified in the investigated genomes in terms of: gene name, corresponding BacMet ID, gene product, gene family, targeted biocide or metal compound, gene description, presence in the core BacMet (i.e. shared by ≥90% of samples), presence in the core of *S.* Napoli, *S.* Typhi and *S.* Paratyphi A subgroup, presence in the core of the non-typhoidal *Salmonella* subgroup, presence in at least two samples of *S.* Napoli, *S.* Typhi and *S.* Paratyphi A subgroup, presence in at least two samples of the non-typhoidal *Salmonella* subgroup.

**Additional file 11: Figure S7.** BacMet hierarchical tree. BacMet genes presence/absence profiles in the investigated genomes where grouped by hierarchical clustering based on Jaccard distance [85] and Ward agglomeration method [86, 87]. BacMet profiles divide isolates into two main subgroups: one, including 24 different BacMet profiles and grouping together *S.* Napoli, *S.* Typhi and *S.* Paratyphi A genomes; the other one, including 53 different BacMet profiles and grouping together all the non-typhoidal genomes. The two main subgroups differ mainly by the absence of *gesAB* and *golTS* in the *S.* Napoli, *S.* Typhi and *S.* Paratyphi A genomes.

**Additional file 12: **Tabular data. Top GO hits. Additional file 11 reports the top 10 enriched GO terms, detailed for each of the three GO categories, for both *S.* Napoli genomes only vs the whole dataset.

**Additional file 13: **Tabular data. COG characterization of unshared/shared genes between typhoidal/non typhoidal genomes and Napoli genomes. Additional file 12 contains the genes found in *S.* Napoli genomes only, in typhoidal serovar genomes only, in non-typhoidal serovars genome only as well as genes shared by *S.* Napoli genomes and typhoidal serovar genomes or genes shared by *S.* Napoli genomes and non-typhoidal serovar genomes. Each gene is characterized by the gene name, the description of its annotation, the number of genomes it was found in, the COG ID number, the COG category, its name and description, as well as the protein ID identified by Prokka, the gene length in aa, the Uniprot Best match and the associated GO terms. Genes annotated as “Hypotetical protein” are filtered from the default view.

**Additional file 14: Fig.ure S8.** Boxplot of detected pseudogenes distribution. Pseudogene content of *S.* Napoli genome was compared to two well known host-generalist serovars (*S*. Enteritidis and *S*. Typhimurium) and three host-restricted serovars (*S.* Typhi, *S.* Paratyphi A and *S*. Gallinarum). Both total number of identified pseudogenes (Panel A) and percentage of pseudogenes over total amount of annotated genes (Panel B) are reported. Kruskal Wallis rank sum test confirmed that a significant difference could be found in pseudogene number distribution among different serovars (*p*-value < 2.2e-16). The number of pseudogenes detected in *S*. Napoli is lower if compared to serovars known to be host-restricted as *S*. Gallinarum (adjusted p-value = 5.143517e-04), *S*. Typhi (adjusted p-value = 2.281705e-19), and *S*. Paratyphi A (adjusted *p*-value = 4.411131e-04).

**Additional file 15 **Tabular data. Prophage hit description. Number of intact/incomplete/questionable prophages and their name, for each sample. In addition, serotype, ST and subclade (for *S.* Napoli genomes only) metadata are reported to ease data investigation.

**Additional file 16: Figure S9.** Heatmap of intact prophage presence/absence in all Clade A investigated genomes. Prophage presence (green) and absence (black) in the investigated genomes is represented in a heatmap. The y axis reports the names of the identified intact prophage sequences; the x axis reports *Salmonella* genomes, grouped by serovar for ease of interpretation. Globally, *S*. Napoli isolates reported a significantly lower number of prophages than other serotypes (Wilcoxon rank sum test, *p*-value < 2.2e-16). P88 prophage was found in 45 *S.* Napoli genomes, thus resulting the most frequently prophage detected in this serovar.

## Abbreviations

ARG: Antimicrobial resistance gene
BfR: German Federal Institute for Risk Assessment
BP: Biological processes
CC: Cellular Component
CDS: Coding sequence
COG: Clusters of Orthologous Groups
DTU: Technical University of Denmark
ENGAGE: Establishing Next Generation Sequencing Ability for Genomic Analysis in Europe
ESBL: Extended-spectrum β–lactamases
GC%: GC-content (or guanine-cytosine content)
GO: Gene Ontology
ID: Identifier
iNTS: Invasive non-typhoidal serovars
ISS: Istituto Superiore di Sanità
IZSVe: Istituto Zooprofilattico Sperimentale delle Venezie
Mb: Mega base pairs = 1,000,000 bp
MDR: Multidrug resistance
MF: Molecular Function
ML: Maximum Likelihood
MLST: Multi Locus Sequence Typing
MRCA: Most Recent Common Ancestor
N50: Sequence length of the shortest contig at 50% of the total genome length
NTS: Non-typhoidal serovars
QAC: Quaternary ammonium compounds
RASFF: Rapid Alert System for Food and Feed
SPIs: *Salmonella* Pathogenicity Islands
ST: Sequence Type
