# Supplementary figures and images for: Comparative genomic analysis reveals high intra-serovar plasticity within Salmonella Napoli isolated in 2005–2017
Source: BMC Genomics. 2020 Mar 4;21:202. doi: 10.1186/s12864-020-6588-y (PMC7057659; doi:10.1186/s12864-020-6588-y)

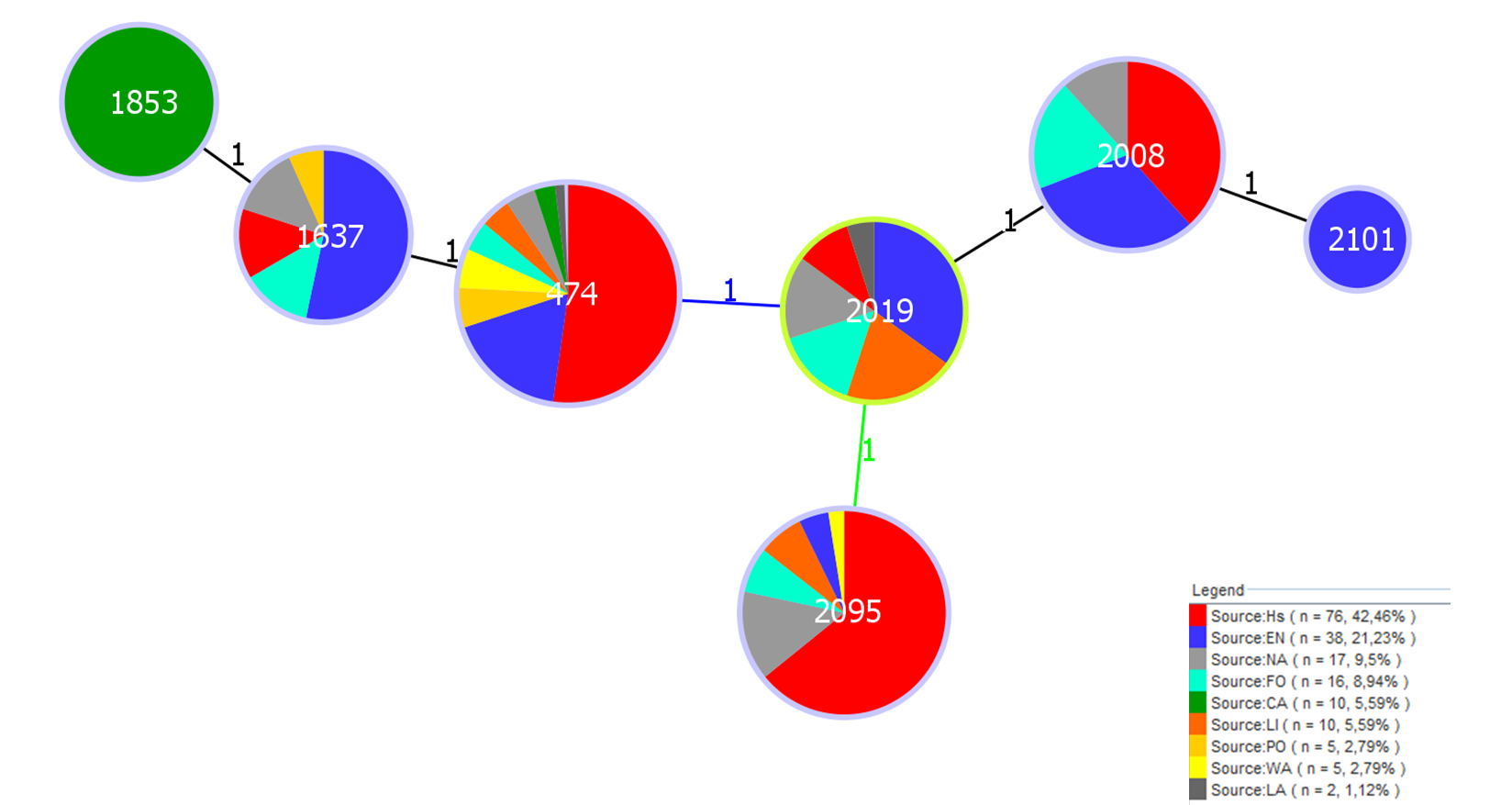

Supplement: Supplementary file 3 — Additional file 3: Figure. S1. MLST minimum-spanning tree of all S. Napoli genomes. Core genome MLST profiles of all S. Napoli isolates was used to build a minimum spanning tree with pie charts describing sources of isolation found for each ST. S. Napoli shows a high variability of sources contributing to each ST, with the exception of ST-1853, related to a single outbreak occurred in Italy in 2012 and associated to kennel dogs [17], and ST-2101 that comprises one sample only. Hs: Homo sapiens, EN: Environment, FO: Food, CA: Companion Animal, LI: Livestock, PO: poultry, WA: Wild Animal, LA: laboratory. [file 12864_2020_6588_MOESM3_ESM.png]

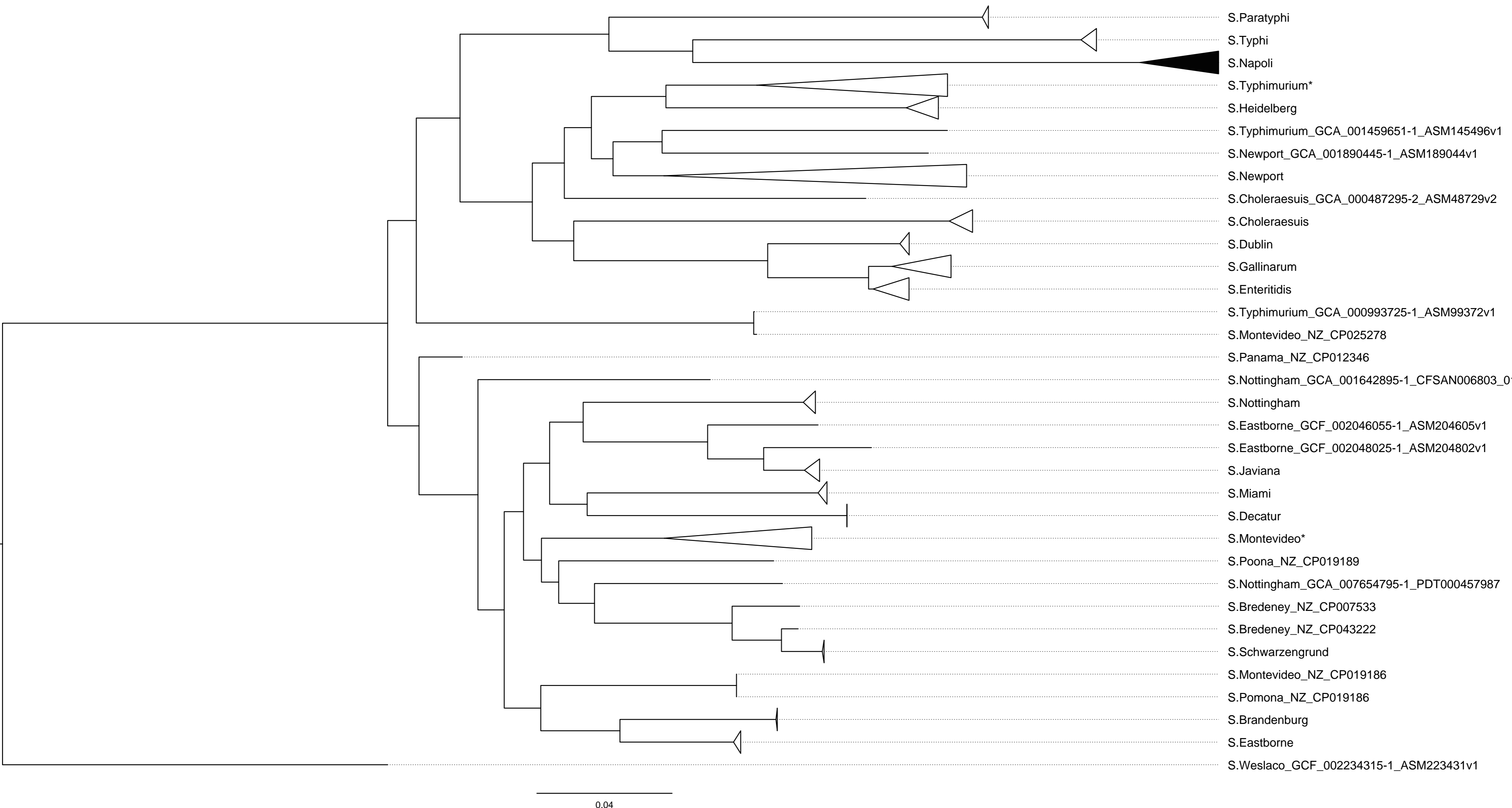

Supplement: Supplementary file 4 — Additional file 4: Figure S2. Core genome alignment-based ML phylogeny of Salmonella serovars belonging to Clade A, B, and C. Core genome alignment was used for phylogenetic reconstruction kSNP3 3.0 [71], with a k-mer lenght of 21. The phylogenetic tree was derived using FastTree [72], with default parameters for nucleotide input (GTR-GAMMA model of nucleotide substitution). Subtrees were collapsed for ease of interpretation. S. Napoli clusters within Clade A, in the typhoidal subclade. [file 12864_2020_6588_MOESM4_ESM.pdf]

K=5

K=6

K=7

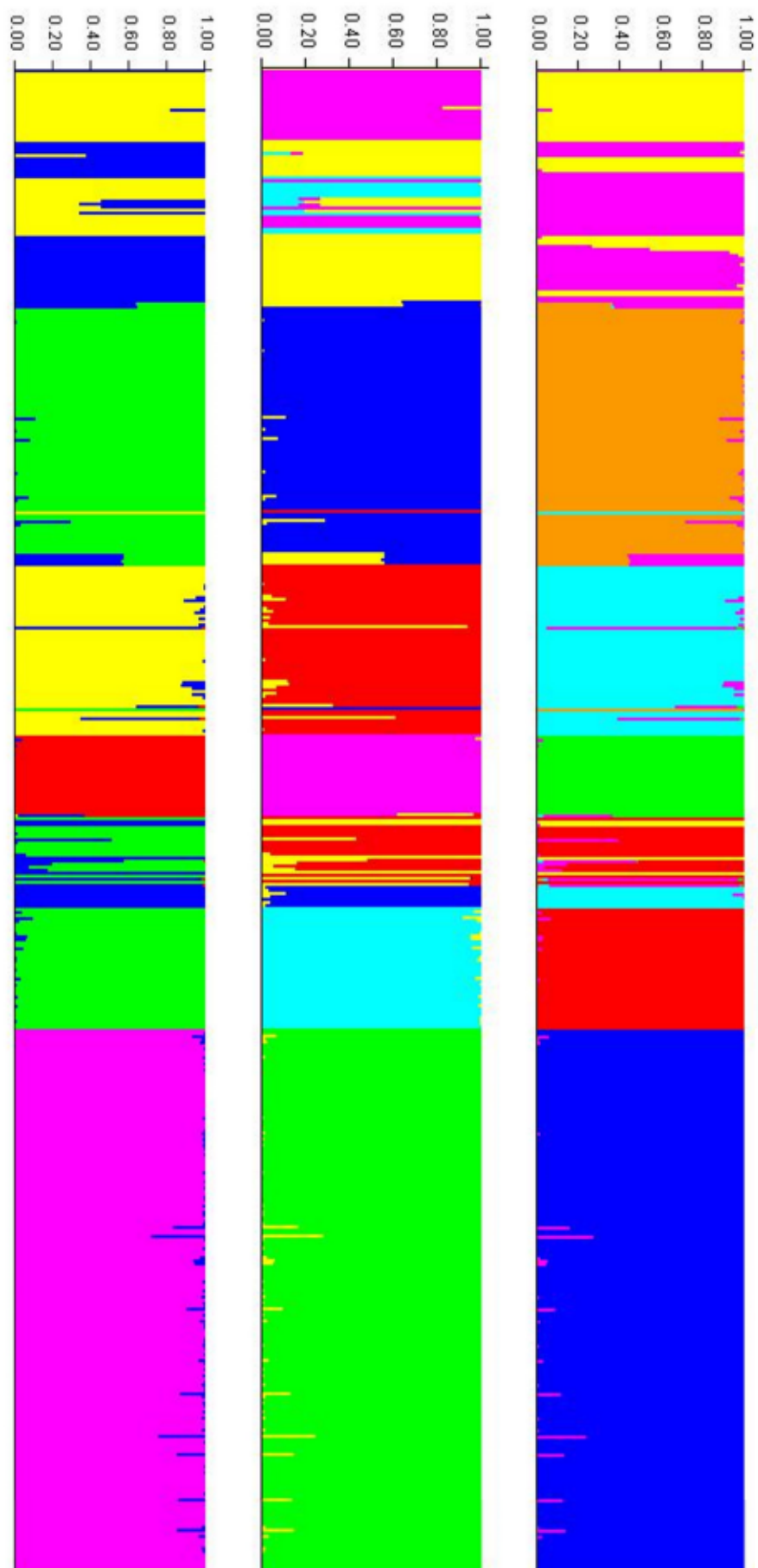

S. Napoli

Supplement: Supplementary file 5 — Additional file 5: Figure S3. Population recombination analysis of serovars belonging to Clade A, B, and C. Core-genome MLST (cgMLST) matrix, including 3065 alleles, trained with Salmonella enterica gene prediction model file, was used as input for STRUCTURE 2.3.3 [26]. A model-based Bayesian clustering method was used to cluster samples into groups. Samples generally clustered in accordance to the phylogenetic analysis (Fig. S2). Interestingly, in every analyzed population (K = 2 to 10), S. Napoli isolates always group together in a single population, thus highlighting that S. Napoli isolates do not show recombination with other serovars. [file 12864_2020_6588_MOESM5_ESM.pdf]

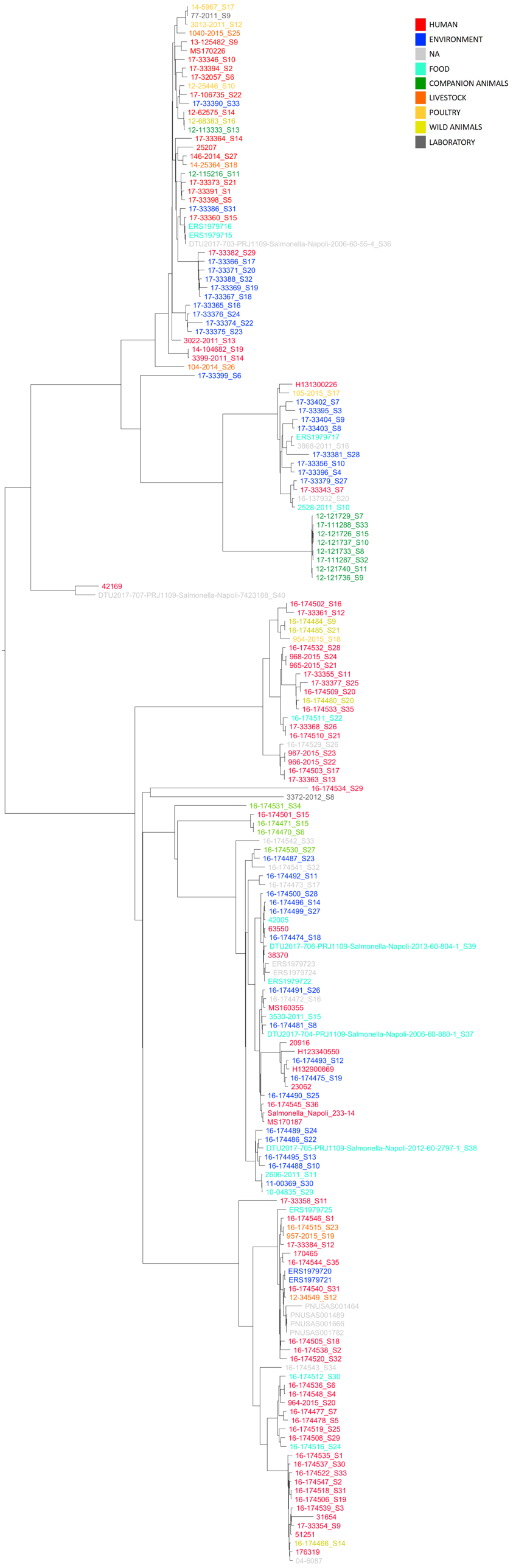

- HUMAN
- ENVIRONMENT
- NA
- FOOD
- COMPANION ANIMALS
- LIVESTOCK
- POULTRY
- WILD ANIMALS
- LABORATORY

Supplement: Supplementary file 7 — Additional file 7 Figure S5. Core genome alignement-based ML phylogeny of S. Napoli genomes. Core genome alignment was used for phylogenetic reconstruction using RAxML (version 7.2.8, [27]) with bootstrapping and Maximum Likelihood (ML) search under the GAMMA model of rate heterogeneity. Tree visualization was obtained using FigTree v1.4.4 [28]. Within S. Napoli serovar, samples cluster primarily by ST, and divide into two major subclades. No significant correlation could be found between phylogenetic clustering and source of isolation. Here source of isolation is shown as a colour scale. [file 12864_2020_6588_MOESM7_ESM.pdf]

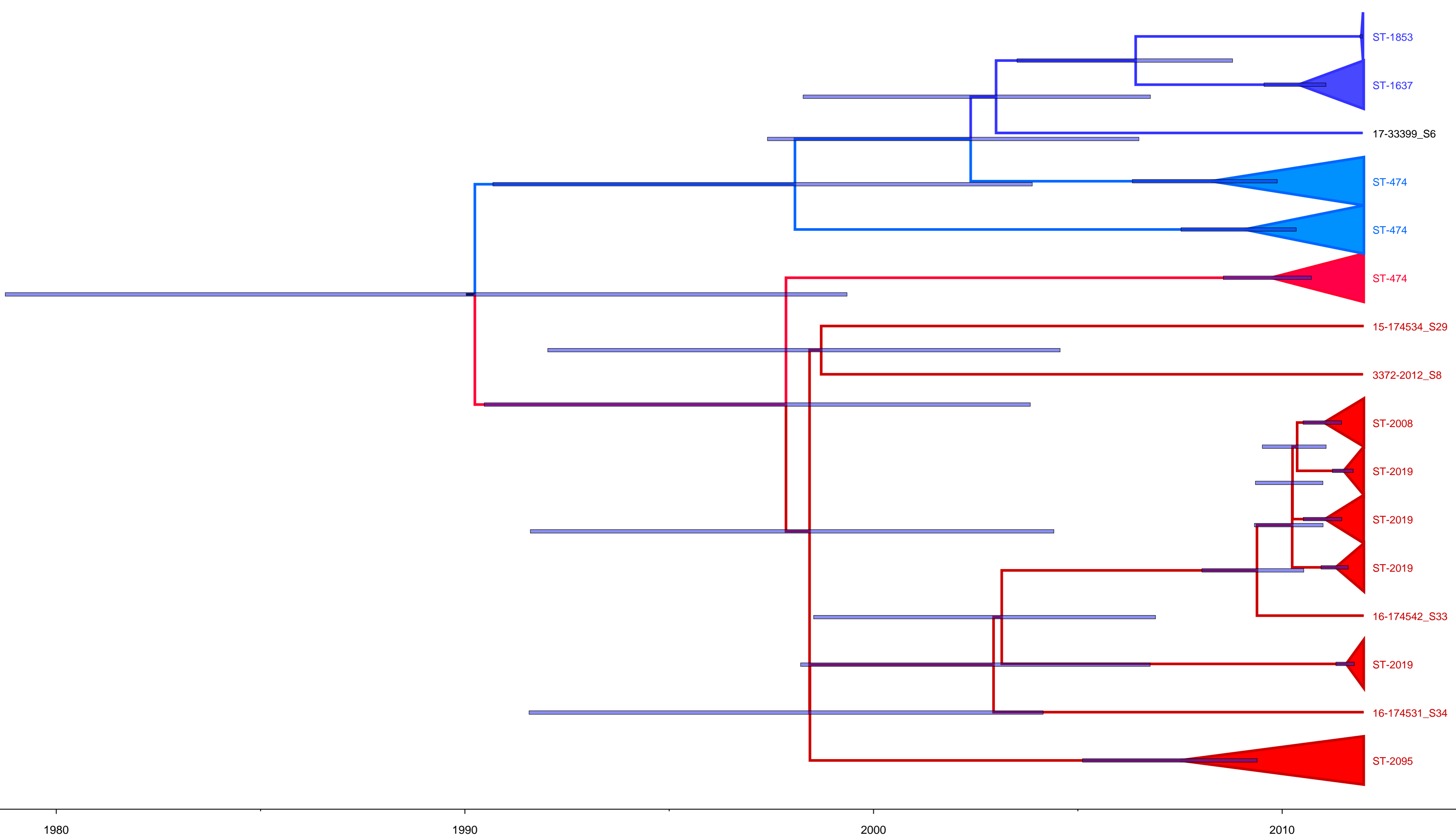

Supplement: Supplementary file 8 — Additional file 8 Figure S6. Bayesian phylogenetic analysis. S. Napoli STs divergence investigation was performed using BEAST [75]. The combination of strict molecular clock model and coalescent log normal population size prior was used. A mean genome-wide corrected evolutionary rate of 8.90 × 10^–8^ sub/site/year (credibility interval: 4.87 × 10^–8^, 13.10 × 10–8) was estimated starting from a 19,496 SNPs matrix built with kNP3 3.0 [71] and indicates that two ST-474 clones might have diverged around 1990. The branches of the maximum clade credibility (MCC) tree are color-coded for comparability with Fig. 2. The scale at the bottom of the tree correspond to calendar years. [file 12864_2020_6588_MOESM8_ESM.pdf]

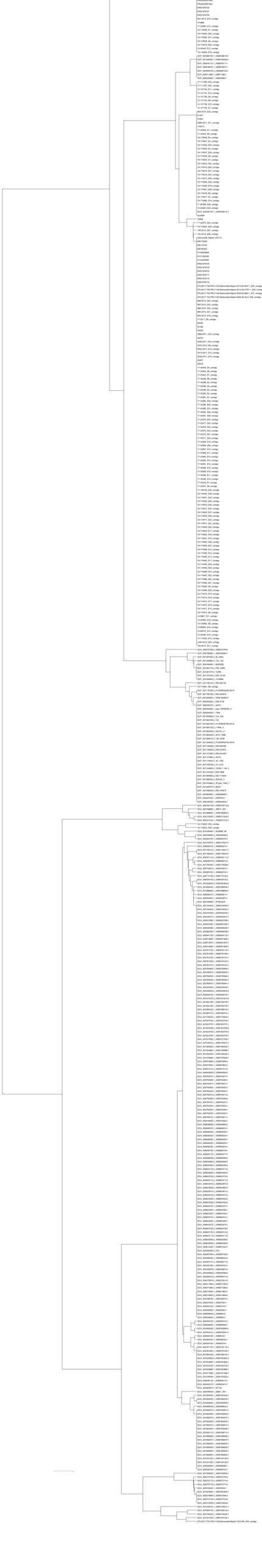

Supplement: Supplementary file 11 — Additional file 11: Figure S7. BacMet hierarchical tree. BacMet genes presence/absence profiles in the investigated genomes where grouped by hierarchical clustering based on Jaccard distance [85] and Ward agglomeration method [86, 87]. BacMet profiles divide isolates into two main subgroups: one, including 24 different BacMet profiles and grouping together S. Napoli, S. Typhi and S. Paratyphi A genomes; the other one, including 53 different BacMet profiles and grouping together all the non-typhoidal genomes. The two main subgroups differ mainly by the absence of gesAB and golTS in the S. Napoli, S. Typhi and S. Paratyphi A genomes. [file 12864_2020_6588_MOESM11_ESM.pdf]

**A**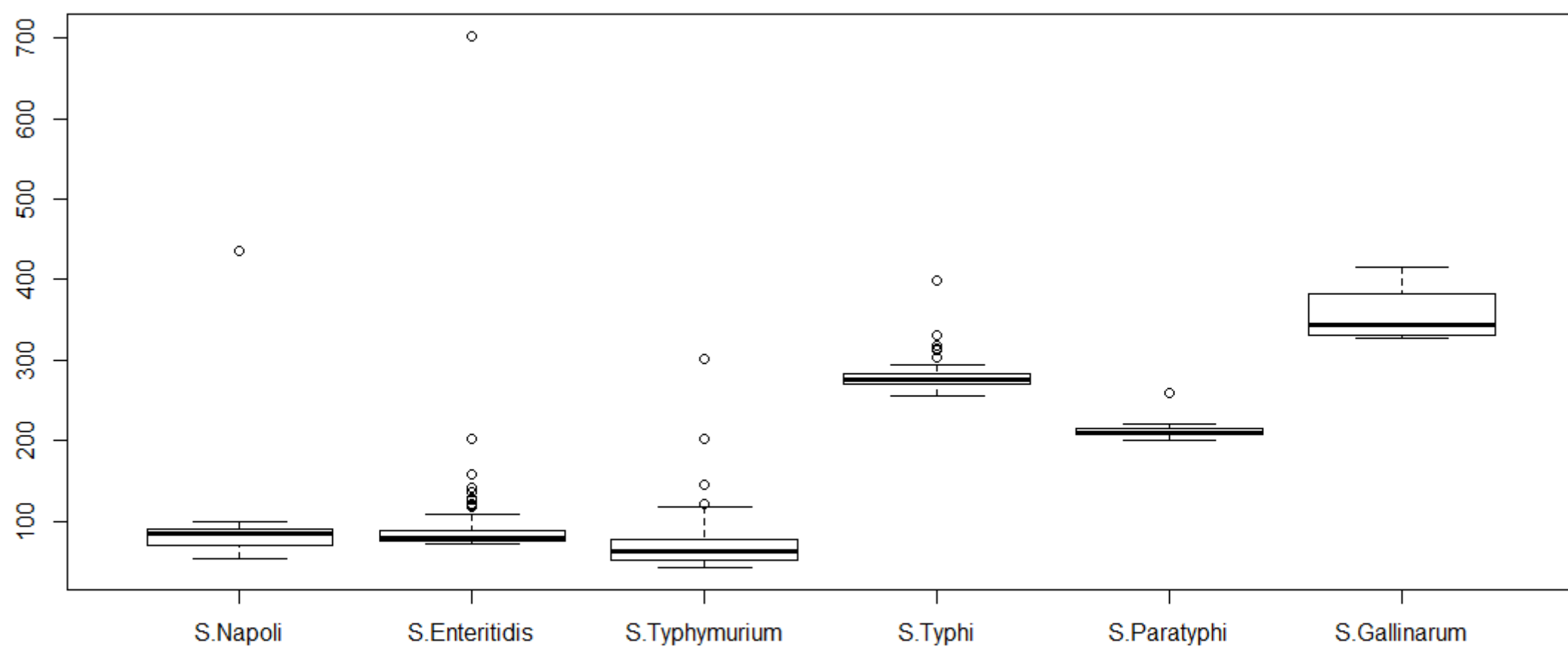**B**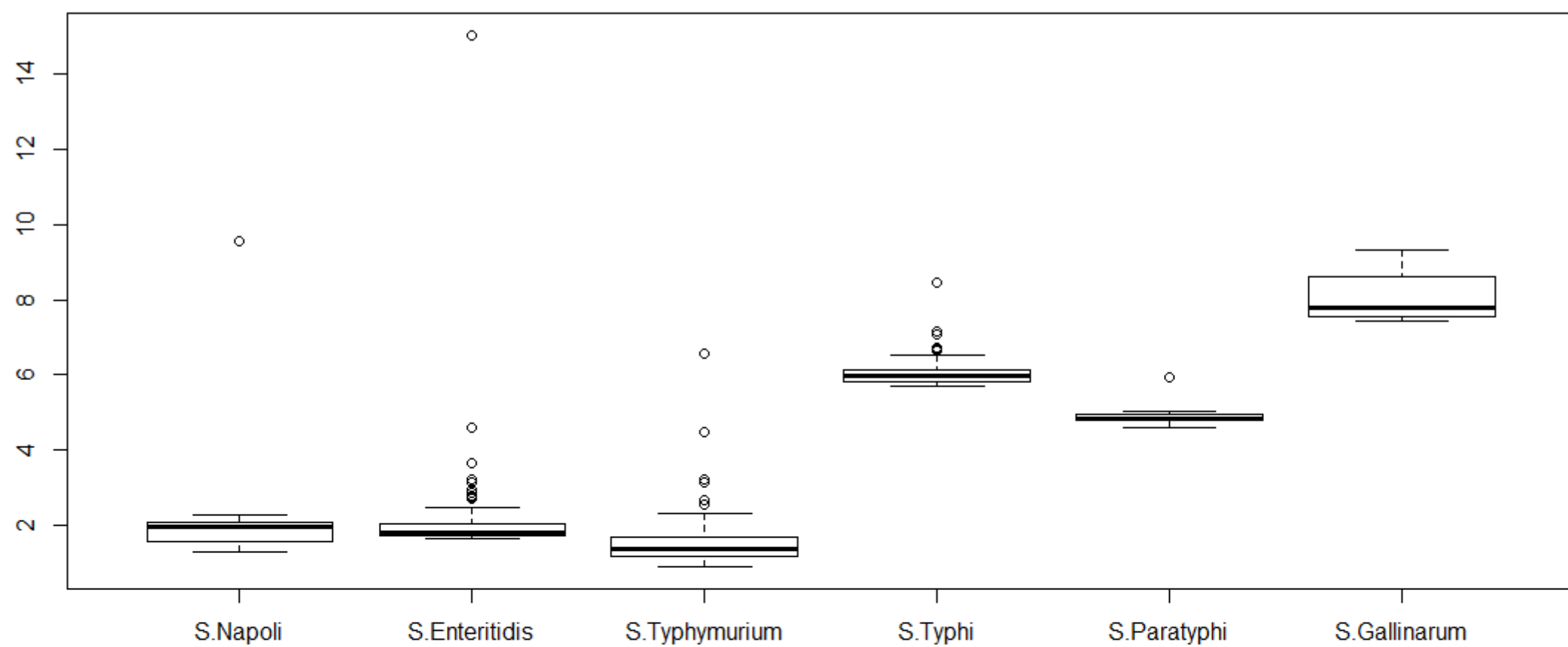

Supplement: Supplementary file 14 — Additional file 14: Fig.ure S8. Boxplot of detected pseudogenes distribution. Pseudogene content of S. Napoli genome was compared to two well known host-generalist serovars (S. Enteritidis and S. Typhimurium) and three host-restricted serovars (S. Typhi, S. Paratyphi A and S. Gallinarum). Both total number of identified pseudogenes (Panel A) and percentage of pseudogenes over total amount of annotated genes (Panel B) are reported. Kruskal Wallis rank sum test confirmed that a significant difference could be found in pseudogene number distribution among different serovars (p-value < 2.2e-16). The number of pseudogenes detected in S. Napoli is lower if compared to serovars known to be host-restricted as S. Gallinarum (adjusted p-value = 5.143517e-04), S. Typhi (adjusted p-value = 2.281705e-19), and S. Paratyphi A (adjusted p-value = 4.411131e-04). [file 12864_2020_6588_MOESM14_ESM.pdf]

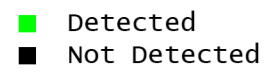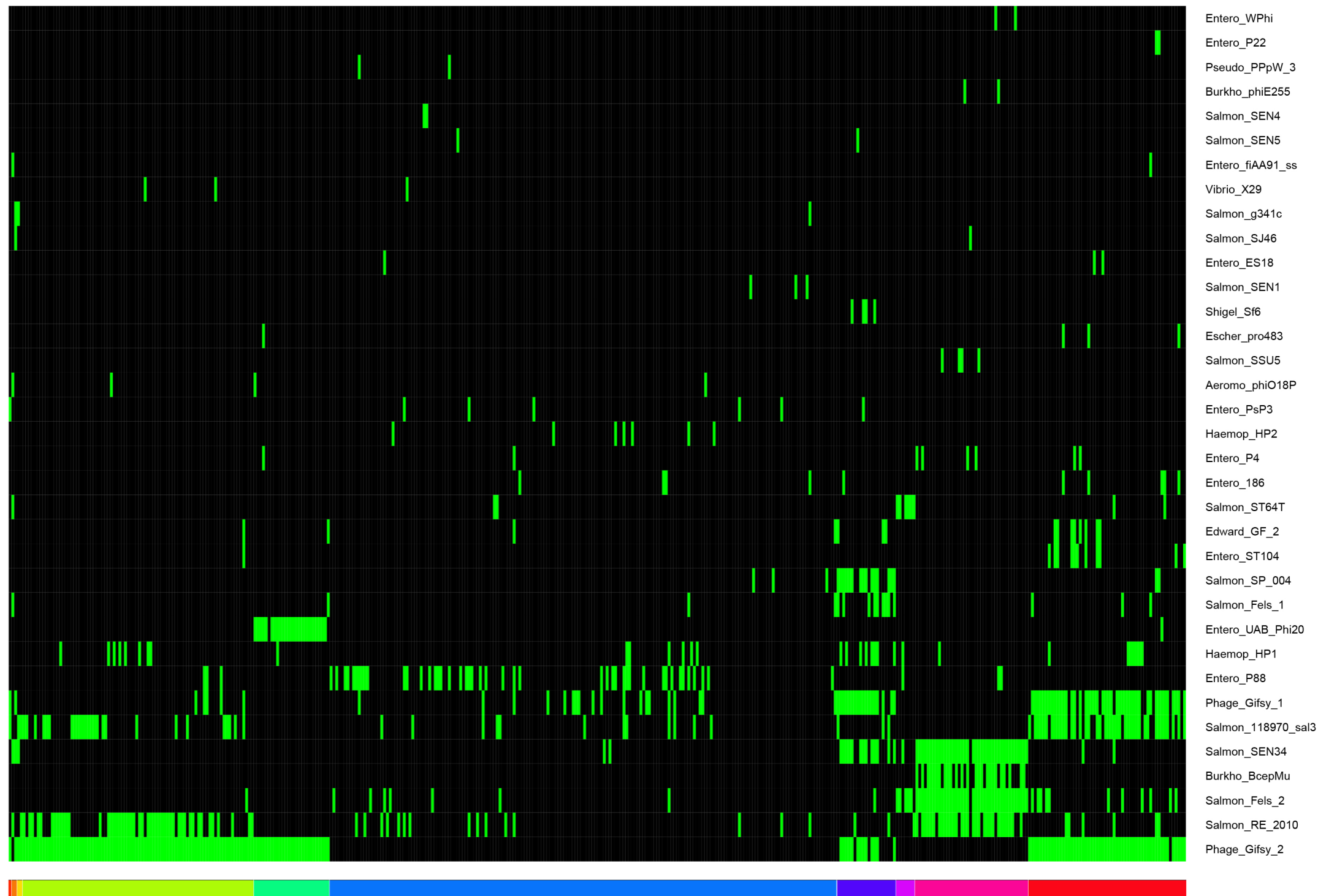

Supplement: Supplementary file 16 — Additional file 16: Figure S9. Heatmap of intact prophage presence/absence in all Clade A investigated genomes. Prophage presence (green) and absence (black) in the investigated genomes is represented in a heatmap. The y axis reports the names of the identified intact prophage sequences; the x axis reports Salmonella genomes, grouped by serovar for ease of interpretation. Globally, S. Napoli isolates reported a significantly lower number of prophages than other serotypes (Wilcoxon rank sum test, p-value < 2.2e-16). P88 prophage was found in 45 S. Napoli genomes, thus resulting the most frequently prophage detected in this serovar. [file 12864_2020_6588_MOESM16_ESM.pdf]
