# Supplementary material for: Comparative genomic analysis reveals high intra-serovar plasticity within Salmonella Napoli isolated in 2005–2017
Source: BMC Genomics. 2020 Mar 4;21:202. doi: 10.1186/s12864-020-6588-y (PMC7057659; doi:10.1186/s12864-020-6588-y)

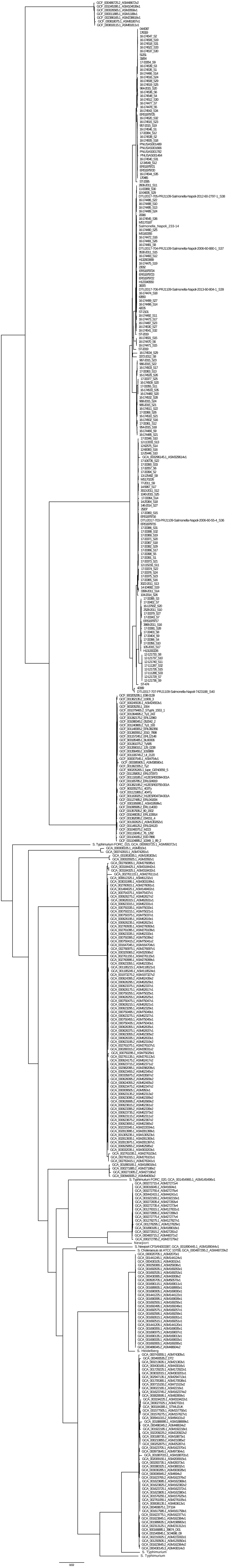

Supplement: Supplementary file 6 — Additional file 6: Figure S4. Core genome alignement-based ML phylogeny of all genomes belonging to Clade A. core genome alignment was used for phylogenetic reconstruction using RAxML (version 7.2.8, [27]) with bootstrapping and Maximum Likelihood (ML) search under the GAMMA model of rate heterogeneity. Tree visualization was obtained using FigTree v1.4.4 [28]. S. Napoli clusters with typhoidal serovars S. Typhi and S. Paratyphi A, separately from all other non-typhoidal serovars. [file 12864_2020_6588_MOESM6_ESM.pdf]
